# Identification of antigens presented by MHC for vaccines against tuberculosis

**DOI:** 10.1038/s41541-019-0148-y

**Published:** 2020-01-03

**Authors:** Paulo Bettencourt, Julius Müller, Annalisa Nicastri, Daire Cantillon, Meera Madhavan, Philip D. Charles, Carine B. Fotso, Rachel Wittenberg, Naomi Bull, Nawamin Pinpathomrat, Simon J. Waddell, Elena Stylianou, Adrian V. S. Hill, Nicola Ternette, Helen McShane

**Affiliations:** 1 Jenner Institute, University of Oxford, Oxford, OX3 7DQ UK; 2 Target Discovery Institute, University of Oxford, Oxford, OX3 7FZ UK; 3 Department of Global Health and Infection, Brighton and Sussex Medical School, University of Sussex, Brighton, BN1 9PX UK

**Keywords:** Vaccines, Tuberculosis

## Abstract

*Mycobacterium tuberculosis* (*M.tb*) is responsible for more deaths globally than any other pathogen. The only available vaccine, bacillus Calmette-Guérin (BCG), has variable efficacy throughout the world. A more effective vaccine is urgently needed. The immune response against tuberculosis relies, at least in part, on CD4^+^ T cells. Protective vaccines require the induction of antigen-specific CD4^+^ T cells via mycobacterial peptides presented by MHC class-II in infected macrophages. In order to identify mycobacterial antigens bound to MHC, we have immunoprecipitated MHC class-I and class-II complexes from THP-1 macrophages infected with BCG, purified MHC class-I and MHC class-II peptides and analysed them by liquid chromatography tandem mass spectrometry. We have successfully identified 94 mycobacterial peptides presented by MHC-II and 43 presented by MHC-I, from 76 and 41 antigens, respectively. These antigens were found to be highly expressed in infected macrophages. Gene ontology analysis suggests most of these antigens are associated with membranes and involved in lipid biosynthesis and transport. The sequences of selected peptides were confirmed by spectral match validation and immunogenicity evaluated by IFN-gamma ELISpot against peripheral blood mononuclear cell from volunteers vaccinated with BCG, *M.tb* latently infected subjects or patients with tuberculosis disease. Three antigens were expressed in viral vectors, and evaluated as vaccine candidates alone or in combination in a murine aerosol *M.tb* challenge model. When delivered in combination, the three candidate vaccines conferred significant protection in the lungs and spleen compared with BCG alone, demonstrating proof-of-concept for this unbiased approach to identifying new candidate antigens.

## Tuberculosis vaccines: immunopeptidomics uncovers potential vaccine antigens

Protective vaccines against *Mycobacterium tuberculosis* (*M.tb*), such as bacillus Calmette-Guérin (BCG), trigger strong CD4 T-cell responses specific to mycobacterium peptides, but their efficacy is variable. Paulo Bettencourt and colleagues now identify a set of mycobacterium peptides presented by BCG-infected macrophages via major compatibility complexes (MHC), and show that three of these antigens can be combined to formulate a vaccine that confers improved protection to Mtb infection in mice. After identifying 94 MHC-II-associated and 43 MHC-I-associated mycobacterium peptides, the researchers performed immunogenicity assays with peripheral blood mononuclear cells from BCG-vaccinated donors, latent Mtb-infected patients and patients with tuberculosis, and show that a set of these peptides was recognised by the immune cells, validating their potential as possible components for new Mtb vaccine formulations. These findings further support the value of immunopeptidomics for the identification of new antigens for effective vaccine alternatives.

## Introduction

*Mycobacterium tuberculosis* (*M.tb*), the etiologic agent of tuberculosis (TB) is the largest cause of death by an infectious disease worldwide. *M. africanum*, *M. canettii*, *M. microtti*, and *M. bovis*, share 99.9% similarity at the nucleotide level and are grouped in the *Mycobacterium tuberculosis* complex (MTC), the mycobacteria causing TB disease. According to the last World Health Organization report, 1.6 million people died of TB, 300,000 of which were co-infected with HIV, in 2017^1^ With the emergence of multi-drug and extensively-drug resistant strains, as well as co-infection with HIV, new tools to control this epidemic are urgently required. The currently available vaccine against TB is a live attenuated form of *Mycobacterium bovis*, strain bacillus Calmette-Guérin (BCG), which has variable efficacy throughout the world.^2^ The immune response against TB relies on CD4^+^ T cells, and to some extent to CD8^+^ T cells,^3^ therefore protective vaccines require the induction of antigen-specific T cells through peptides presented by MHC-II and MHC-I, respectively, in infected macrophages. The identification of MTC antigens presented by MHC molecules in infected macrophages will facilitate vaccine development to boost the protective efficacy induced by BCG vaccination.

Even though CD4^+^ and CD8^+^ T cells can secrete IFN-gamma (IFNγ) upon recognition of antigen, the lack of stringent immunological markers that serve as correlates of protection makes it difficult to predict whether the immune response induced by a vaccine will result in protective efficacy.^4^ Currently there are more than a dozen vaccine candidates in different stages of clinical trials including subunit vaccines delivered by viral vectors, proteins in adjuvant, killed mycobacterial whole cell or extracts, recombinant BCG expressing *M.tb* genes, attenuated *M.tb* strains and BCG revaccination strategies.^5^ Although a multitude of platforms are currently being explored for the delivery of antigens designed to replace or boost BCG, current subunit vaccines only use a limited selection of antigens.^6^

Recent advances in immunopeptidomics based on improvements in mass spectrometry instrumentation and data analysis have led to an unprecedented improvement in sensitivity. It is now possible to precisely identify peptide sequences, bound to MHC molecules, at the femto molar scale.^7,8^ This technology allowed the identification of epitopes presented by conventional HLA class-I molecules in ovarian cancer,^9^ influenza,^10^ hepatitis C,^11^ HIV,^12^ and TB.^13^ Unconventional class-I, HLA-E bound peptides have been identified in cells infected with *M.tb*.^14^ Leishmania HLA class-II bound peptides have been also identified, and vaccines developed based on these antigens.^15^ Immunopeptidomics studies have attempted to decipher the immunopeptidome of mycobacteria-infected cells. Only a handful of MHC-I peptides have been described thus far^13^ and MHC-II peptides have not been yet identified. The identification of new vaccine antigen candidates designed to boost BCG has been limited by inherent difficulties of culturing mycobacteria and by the paucity of antigens identified so far using conventional methods. Moreover, the ability of pathogenic mycobacteria to downregulate antigen processing and presentation limits the ability to identify peptides presented by MHC molecules.

Here we have used immunopeptidomic based identification of peptides presented by MHC-I and MHC-II in macrophages infected with *M. bovis* BCG, applying an immunopeptidomics pipeline for peptide identification by mass spectrometry and bioinformatics^8^ (Supplementary Fig. 1). THP-1 cells were selected for this study because these are the most well-characterised human macrophage cell line with a defined HLA generic genotype HLA-A*02:01, HLA-B*15:11, HLA-B*15:15, HLA-C*03:03, HLA-C*03:13, HLA-DRB1*01:01, HLA-DRB1*15:01, HLA-DRB5*01:01, HLA-DQB1*06:02, HLA-DQB1*05:01, HLA-DPB1*04:02 and HLA-DQP1*02:01 (Supplementary Table 1 for allelic details), which is required for peptide binding prediction analysis once peptides have been identified. To overcome the ability of pathogenic mycobacteria to downregulate antigen processing and presentation, we stimulated cells with a cytokine mix to induce higher MHC class-II presentation, immunoprecipitated both MHC-I and MHC-II-peptide bound complexes and analysed by mass spectrometry, leading to the identification of mycobacterial peptides presented by both MHC-I and MHC-II. We have successfully identified 94 mycobacterial peptides presented by MHC-II and 43 presented by MHC-I, from 76 and 41 antigens, respectively. We have mapped the gene expression of BCG in infected macrophages and correlated the expression of the antigens identified with the global gene expression pattern in vivo. Finally, three antigens were selected, expressed in viral vectors and evaluated as vaccine candidates in a murine aerosol *M.tb* challenge experiment. The three candidate antigens, when delivered as viral vectors to boost previous BCG vaccination, conferred significant protection in the lungs and spleen of mice when administered in combination compared to BCG alone. This demonstrates proof-of-concept for this unbiased approach to identify new candidate antigens required for TB vaccine development.

## Results

### Immunopetidomics pipeline can be used to identify BCG-derived peptides presented by MHC molecules

To maximise identification of BCG peptides presented by THP-1 cell MHC molecules, a range of conditions were performed across four infection experiments (Table 1). In all experiments, THP-1 cells were differentiated into macrophages and infected with BCG-GFP. The two first experiments consisted of 2.5 x 10^8^ cells infected with BCG-GFP, macrophages were harvested at 1 and 7 days post-infection. In the first experiment an immunoprecipitation against MHC-I was performed while in the second experiment both MHC-II and MHC-I immunoprecipitations were conducted (Table 1).

**Table 1:** Description of the samples.

	Exp 1	Exp 2	Exp 3	Exp 4
Number of cells per sample	2.5 × 10^8^	2.5 × 10^8^	5 × 10^7^	5 × 10^8^
Rate of infection (%)	30	30	40	21
Culture flasks	T1000	T175	T175	T175
Cytokine pre-treatment	No	No	Yes	Yes
Heat Killed BCG	No	No	Yes	Yes
Immunoprecipitation	MHC-I	MHC-I and -II	MHC-I and -II	MHC-I and -II
Number of samples	3	3	6	6
Number of total replicates	6	9	12	22
Name of samples	CONTROL (A, B)	CONTROL (A, B)^a^	NOCYT NOINF	NOCYT NOINF (A, B)
(replicates A/B)	DAY 1 (A, B)	DAY 1 (A, B)^a^	NOCYT HKBCG	NOCYT HKBCG (A, B)
	DAY 7 (A, B)	DAY 7 (A, B)^a^	NOCYT LIVEBCG	NOCYT LIVEBCG (A, B)
			CYT NOINF	CYT NOINF (A, B)
			CYT HKBCG	CYT HKBCG (A,B)^a^
			CYT LIVEBCG	CYT LIVEBCG (A, B)^a^

^a^Samples with two replicates for MHC-I and one replicate for MHC-II

Pathogenic mycobacteria are known to downregulate antigen presentation^16^ at the gene expression level,^17^ at the antigen processing level^17,18^ and at the antigen presentation level,^18^ therefore limiting our ability to identify peptides. To overcome this, we developed a cytokine cocktail to improve the expression of MHC-II molecules at the surface of infected macrophages. Interleukin-10 (IL10) is secreted by macrophages infected with pathogenic mycobacteria^18^ and likely acts as an anti-inflammatory molecule reducing antigen processing and presentation. IFNγ has been shown to improve gene expression of MHC-II genes in infected THP-1 cells.^17^ TNFα is important in the control of TB through diverse mechanisms including macrophage activation and granuloma formation.^19^ We tested the effect of IFNγ, TNFα and anti-IL10 antibodies, alone or in combination to enhance MHC-II presentation in macrophages infected with BCG, and we were able to improve MHC-II but not MHC-I presentation (Supplementary Fig. 2b, c). We observed that IFNγ alone was able to induce higher expression of HLA-DR but combining with other cytokines did not produce a synergistic effect. Furthermore, as live mycobacteria are known to actively block antigen processing and presentation^17,18^ we also treated macrophages with heat-killed BCG-GFP (HK-BCG). For the third and fourth experiments, we prepared six samples, three of which were stimulated with cytokine cocktail containing IFNγ to improve MHC-II expression and anti-IL10 to prevent the reduced antigen processing and presentation caused by mycobacterial infection, and three of which were left untreated. The three samples in each of these groups were then infected with live BCG-GFP, treated with HK-BCG or left uninfected. In the third experiment each sample was composed of 5 × 10^7^ cells while in the fourth experiment each sample contained 5 × 10^8^ cells. The rate of infection in all experiments ranged between 21 and 40% (Table 1 and Supplementary Fig. 2a).

### Mycobacterial peptides are presented by MHC-I and MHC-II in infected macrophages

**Fig. 1:**
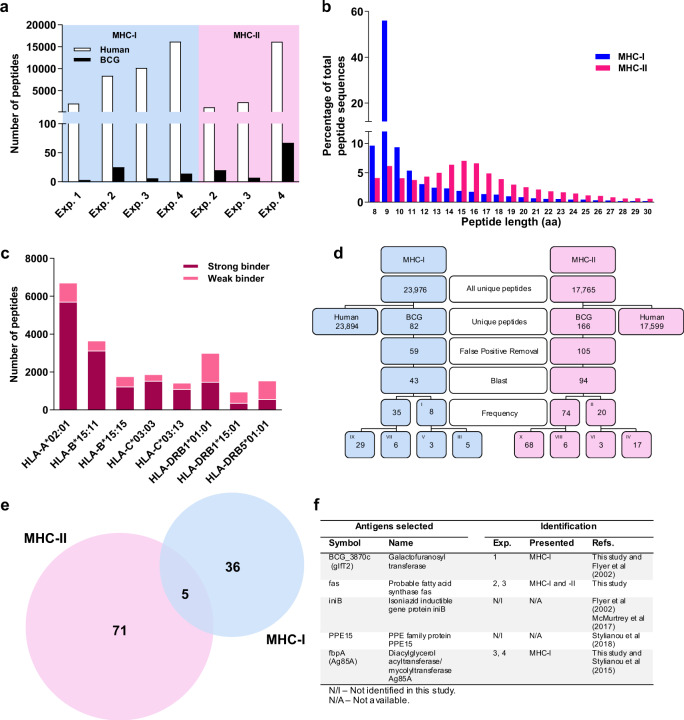
Peptide identification and selection of vaccine candidates. **a** Distribution of unique human and BCG peptides identified in each experiment. Distribution of unique human peptides from the four experiments combined, identified in function of length (**b**), and according to the netMHCpan rank (**c**). The best match HLA binding allele for each peptide was determined and selected. MHC-I peptides with a rank ≤ 0.5 or between 0.5 and 3.0 were considered strong or weak binders, respectively. MHC-II peptides with a rank ≤ 2 or between 2 and 10 were considered strong or weak binders, respectively. The remaining peptides were excluded. The alleles identified as best binders were HLA-A*02:01, HLA-B*15:11, HLA-B*15:15, HLA-C*03:03, HLA-C*03:13, HLA-DRB1*01:01, HLA-DRB1*15:01 and HLA-DRB5*01:01. **d** Selection of unique BCG peptides from total peptides following the pipeline for identification. A Peaks score cut-off of >15 was used for the peptide identification. After applying our data analysis pipeline, peptides were selected for presence in more than 1 sample (I, II). These were further selected if found in different experiments (III, IV) or if found in more than one sample but not in different experiments (V, VI). If peptides were not present in more than one sample, they were further down-selected into different peptide sequences found on the same antigen (VII, VIII) or found only once (IX, X). MHC-II peptides also presented nested sequences included in II and IV. **e** Total BCG antigens identified by MHC-I and MHC-II. **f** Table showing selected antigens for vaccine production.

Following mass spectrometric analysis of each sample, peptide spectra were analysed using Peaks (Bioinformatics Solutions). The false discovery rate (FDR) of peptide identifications was controlled using in-build simultaneous interrogation of the spectra through a decoy database fused to the target sequence database.^20^ Each spectrum was assigned a Peaks score reflecting the confidence of the sequence interpretation. We applied a Peaks score cut-off of 15 to all samples, resulting in an average FDR of 1.65% for MHC-I, and 10.37% for MHC-II peptides. We identified a total number of 23,976 unique MHC-I and 17,765 unique MHC-II peptides. From these, 23,894 MHC-I human, 43 MHC-I BCG, 17,599 MHC-II human and 94 MHC-II BCG peptides (Fig. 1a, d).

The MHC-I peptides showed a typical distribution of amino acid length of all human peptides identified, with the predominant 9-mer peak consisting of 55.98% of all MHC-I peptides (Fig. 1b). The distribution of the MHC-II peptides revealed an enrichment of peptides ranging from 12 to 18 amino acids long (Fig. 1b). Interestingly, a 9-mer peak can be observed within the MHC-II peptides, reflecting a potential non-specific co-precipitation of MHC-I peptides with MHC-II peptides^21^ (Fig. 1b). BCG-derived peptides were further selected based on their amino acid length. From a total of 82 MHC-I and 166 MHC-II BCG unique peptides, all MHC-I BCG peptides were 8–15 amino acids and all MHC-II BCG peptides were 8–25 amino acids in length, (Fig. 1d). False positive sequences were excluded and 59 MHC-I and 105 MHC-II BCG peptide sequences were further analysed (Fig. 1d). These sequences were further Blast analysed using the DeBosT script developed for this study. Moreover, the amino acids leucine (L) and isoleucine (I) are isomers, which are indistinguishable from each other in our mass spectrometry protocol. All peptides were blasted for all possible combinations of I and L, and when a combination matched a human peptide, the sequence was excluded. Applying these four data analysis criteria, we have identified 43 BCG MHC-I and 94 BCG MHC-II peptides (Fig. 1d).

Forty-one MHC-I and 76 MHC-II BCG antigens were identified from the peptide sequences. Interestingly, five BCG antigens were common to both MHC-I and MHC-II (Fig. 1e and Table 2). The peptides from the common antigens identified in MHC-II had an amino acid length ranging from 9 to 13, suggesting these could be MHC-I peptides that co-precipitated unspecifically with the MHC-II immunoprecipitation, rather than being the product of antigens presented by both MHC-I and MHC-II.

**Table 2:** Antigens identified in MHC-I, -II or both.

Antigen identification					
MHC-I		MHC-II		MHC-I and -II	
Symbol	Accession	Symbol	Accession	Symbol	Accession
BCG_0270	A1KF53	BCG_0082	A0A0H3M0K6	carB	A0A0H3MCS3
BCG_0405c	A0A0H3M1D1	BCG_0116	A0A0H3M793	dxs	A1KM20
BCG_1061c	A0A0H3M9A8	BCG_0234	A0A0H3MA06	fas	A0A0H3MFR6
BCG_1241	A0A0H3M5F4	BCG_0323	A0A0H3M155	PE_PGRS27	A0A0H3M617
BCG_1242c	A0A0H3MC94	BCG_0465c	A0A0H3M812	PPE8	A0A0H3M207
BCG_2410	A0A0H3MFF6	BCG_0526	A0A0H3MAK2		
BCG_2427c	A0A0H3MFH1	BCG_0845c	A0A0H3M332		
BCG_2476c	A0A0H3M6D2	BCG_0897	A0A0H3M2H3		
BCG_2532c	A0A0H3MCT0	BCG_1381c	A0A0H3MA33		
BCG_2582c	A0A0H3MFU7	BCG_1493	A0A0H3M602		
BCG_2830	A0A0H3M7I5	BCG_1520c	A0A0H3M3X1		
BCG_3501c	A0A0H3M9J8	BCG_1721	A0A0H3M4I3		
BCG_3864c	A0A0H3MAQ0	BCG_2160	A0A0H3M5I7		
BCG_3870c	A0A0H3MAQ2	BCG_2240c	A0A0H3M833		
BCG_3967	A0A0H3MCA7	BCG_2938	A0A0H3M9W9		
bioB	A1KJ05	BCG_3469	A1KP90		
carB	A0A0H3MCS3	BCG_3644c	A1KPR5		
cydD	A0A0H3MDE4	BCG_3738	A0A0H3MIR0		
dxs	A1KM20	BCG_3788	A0A0H3MBW3		
fadD24	A0A0H3M520	BCG_3838	A0A0H3MAM8		
fas	A0A0H3MFR6	BCG_3906c	A0A0H3MAU4		
fbpA	A1KQD8	BCG_3941c	A0A0H3MC87		
fbpB	A1KJU9	carB	A0A0H3MCS3		
fbpC	A0A0H3M0R3	ctpJ	A0A0H3M9W7		
iniC	A0A0H3M1A8	dipZ	A0A0H3M9U0		
mbtD	A0A0H3M738	dnaB	A0A0H3M178		
metH	A0A0H3M6G7	dnaN_1	A0A0G2Q9D4		
mmpL12	A0A0H3M689	dxs	A1KM20		
mmpL14	A0A0H3M5L6	echA5	A0A0H3M485		
mmpL3	A0A0H3M2S6	efpA	A0A0H3MDJ5		
PE_PGRS14	A0A0H3M376	embA	A0A0H3MFP8		
PE_PGRS27	A0A0H3M617	embB	A0A0H3MC15		
pks12	A0A0H3M7L7	eno	A1KHG1		
PPE24	A0A0H3M5L1	fadD26	A0A0H3M8A6		
PPE4	A0A0H3MA86	fadE34	A0A0H3MIH3		
PPE8	A0A0H3M207	fas	A0A0H3MFR6		
ppsD	A0A0H3MGQ7	ftsY	A0A0H3MDN2		
rpfA	A0A0H3M8Z9	gap	A0A0H3MAB5		
sseB	A0A0H3MF71	gltB	A0A0H3MJE7		
ugpBa	A0A0H3M9Q7	groL2	A1KFR2		
uvrA	A0A0H3M4F7	groS	A1KPA9		
		hbhA	A1KFU9		
		hemE	A1KM16		
		hisD	A0A0H3M564		
		hns	A0A0H3MC68		
		htrA	A0A0H3M5J1		
		hupB	A0A0H3M7X4		
		kgd	A1KI36		
		lipE	A0A0H3MJ54		
		metE	A1KHS4		
		moeA1	A0A0H3M2X4		
		mtc28	A0A0H3M0J7		
		murE	A0A0H3M5J7		
		narK2	A0A0H3M5J6		
		pdhC	A0A0H3M6I2		
		PE_PGRS13	A0A0H3MB82		
		PE_PGRS15	A0A0H3M903		
		PE_PGRS23	A0A0H3M3G6		
		PE_PGRS27	A0A0H3M617		
		PE_PGRS29	A0A0H3M4W3		
		PE_PGRS3	A0A0H3M7N8		
		PE_PGRS50	A0A0H3MHS0		
		PE_PGRS53	A0A0H3MIA6		
		PE_PGRS54	A0A0H3M9Q3		
		PE_PGRS7	A0A0H3M1W6		
		PE_PGRS9	A0A0H3M8Q1		
		pknJ	A0A0H3M6E7		
		pks2	A1KQG0		
		pks8	A0A0H3M4G9		
		PPE28	A0A0H3M5P8		
		PPE50	A0A0H3M8U4		
		PPE55a	A0A0H3M8U0		
		PPE8	A0A0H3M207		
		rplV	A1KGI7		
		rpoB	A1KGE7		
		topA	A0A0H3MA36		

All peptides identified were analysed through netMHCpan 4.0^22^ and netMHCpanII 3.2,^23^ the reference algorithms for both MHC-I and MHC-II peptide prediction, respectively. The best match HLA binding allele and the best match score were identified and plotted for all human peptides across all samples. MHC-I peptides were more abundant that MHC-II peptides. Most MHC-I peptides were strong binders whereas most MHC-II peptides were weak binders (Fig. 1c).

After additional analysis of location, function and intracellular expression level, new candidates were selected based on the highest expression levels, IFNγ-ELISpot responses and Spectral Match Validation, for further analysis as proof-of-concept. From the list of BCG antigens identified (Table 2, Supplementary Tables 2 and 3), three were selected for evaluation as vaccine candidates (Fig. 1f). The peptide BCG_3870c_4-12_ LAASLLSRV from the Galactofuranosyl transferase BCG_3870c has 100% identity to Galactofuranosyltransferase glfT2 from *M.tb* (Rv3808c) and was identified in two samples of the first experiment, as a MHC-I bound peptide.

The fatty acid synthase (fas), was identified associated to both MHC-I and MHC-II molecules. The peptide fas_2248-2257_ ADLVVIVGGA was identified associated to MHC-I in the second experiment. The peptide fas_57–65_ GIETELATL was found associated to MHC-I in the sample NOCYT LIVEBCG from the third experiment, and the peptide fas_241–249_ TPEQLSRFE was found associated to MCH-II in the same sample (Supplementary Tables 2 and 3).

The peptide from Ag85A, fbpA_44–51_ FSRPGLPV, was found associated to MHC-I in the sample CYT HKBCG from the third experiment and samples CYT HKBCG A and B from the fourth experiment. Remarkably, this peptide is also present in Ag85B (fbpB_41–48_ FSRPGLPV) and Ag85C (fbpC_47–54_ FSRPGLPV). Viral vectors expressing Ag85A have been shown to improve the protective efficacy of BCG.^24^ For these reasons, this antigen was selected for vaccine production (Fig. 1f). The iniB and PPE15 antigens were selected because they have been described previously as presented by MHC-I molecules.^3,13^

### Antigens presented by MHC-I and MHC-II are highly expressed in infected cells

**Fig. 2:**
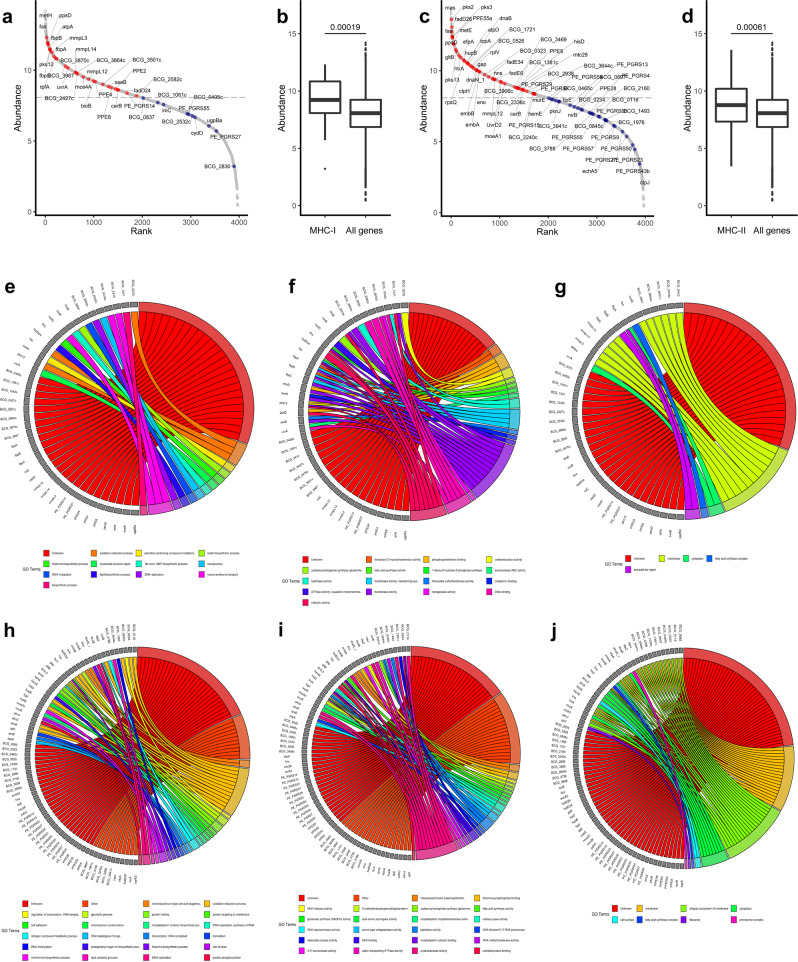
Gene expression and gene ontology of antigens identified. Associated genes of MHC-I (**a**) and MHC-II presented peptides (**c**) are highly expressed in *M. bovis* BCG RNA extracted after 24 h infection of THP-1 cells. Normalised expression levels of *M. bovis* BCG genes were measured by RNA-seq. Genes encoding peptides detected on MHC-I (**b**) or MHC-II (**d**) are significantly higher expressed compared to all genes in *M. bovis* BCG RNA extracted after 24 h infection of THP-1 cells. The lower and upper hinges correspond to the first and third quartiles (the 25th and 75th percentiles) and the centre line corresponds to the median. The upper whisker extends from the hinge to the largest value no further than 1.5 * IQR from the hinge (where IQR is the interquartile range). The lower whisker extends from the hinge to the smallest value at most 1.5 * IQR of the hinge. Gene ontology of identified antigens. Proteins of MHC-I and MHC-II associated peptides (Table 2) were linked to EBI QuickGO Gene ontology clusters and displayed as chord plots for the three aspects Biological Process (**e**, **h**), Molecular Function (**f**, **i**) and Cellular Component (**g**, **j**). Proteins without a GO category were labelled as Unknown. To prevent overplotting, only the first 22 GO categories are shown and all lower categories as determined by enrichment p-values were labelled as “Other”. *P*-values were estimated using a Mann–Whitney test.

To verify whether the antigens identified were expressed in macrophages infected by BCG, we performed RNA-seq on THP-1 cells infected with BCG, 1 day post-infection. Proteins identified from peptides presented by MHC-I and MHC-II were plotted against normalised expression levels of all BCG genes (Fig. 2a, c). Antigens presented by MHC-I were highly expressed compared to all BCG genes (*p* = 0.00019) (Fig. 2b). MHC-II antigens were also highly expressed (*p* = 0.00061) although to a lesser extent (Fig. 2d). The most highly expressed gene presented by MHC-I was fas, followed by BCG_3870c (glfT2) and by all three Ag85-complex genes (fbpABC) and others. The highest expressed gene presented by MHC-II was pks2, followed by mas, fadD26, pks3 and PPE55a.

### Antigens presented by MHC-I and MHC-II are mostly associated with membranes and involved in lipid metabolism

To understand the predicted biological function and the cellular location of each antigen, we mapped the gene ontology of each antigen (Fig. 2e–j). Despite 37% of the antigens having unknown biological functions, 47% having unknown biological processes, and 60% having unknown subcellular locations, the antigens identified on MHC-I cluster mostly in the process of transmembrane transport (Fig. 2e), with transferase activity functions (Fig. 2f) located at the membrane (Fig. 2g). The antigens identified on MHC-II cluster mostly in oxidation-reduction processes and functions (Fig. 2h, i), and were strongly associated to a membrane location (Fig. 2j). Most antigens, with known functions, identified on both MHC-I and MHC-II were associated with the biosynthesis and transport of lipids and were located at the cell membrane, however, there is no significant over or under representation of membrane- or cell wall-associated proteins relatively to the entire BCG proteome.

### Peptide sequences identified by mass spectrometry are confirmed by spectral match validation

Based on the FDR, the PSMs identified using Peaks have a low probability of being false positives. However, to confirm that the peptide sequences were assigned correctly, a spectral match validation experiment was performed. Synthetic peptides were produced and compared to a selection of PSMs obtained from our experiments, herein referred to as biological peptides. Synthetic peptides were run in the same experimental conditions as biological peptides. The mass over charge [m/z] for each peptide, the charge state, the intensity and distribution of each peak within each peptide sequences, as well as the peptide-specific retention time (RT) on the liquid chromatography were compared between synthetic and biological peptides and the sequences matched (Supplementary Fig. 3).

### Single peptides identified by immunopeptidomics are recognised by PBMCs from HLA-matched BCG-vaccinated subjects

To evaluate whether the peptides identified could be recognised by BCG-vaccinated volunteers, IFNγ-ELISpots were performed on peripheral blood mononuclear cells (PBMCs) collected in previous clinical trials. Peptide responses were evaluated in healthy volunteers who had received BCG vaccination, subjects with latent *M.tb* infection and patients with active TB disease. The HLA types of the volunteers were known (Supplementary Table 4), allowing us to match a subset to the HLA type of the THP-1 cells (Supplementary Table 1).

**Fig. 3:**
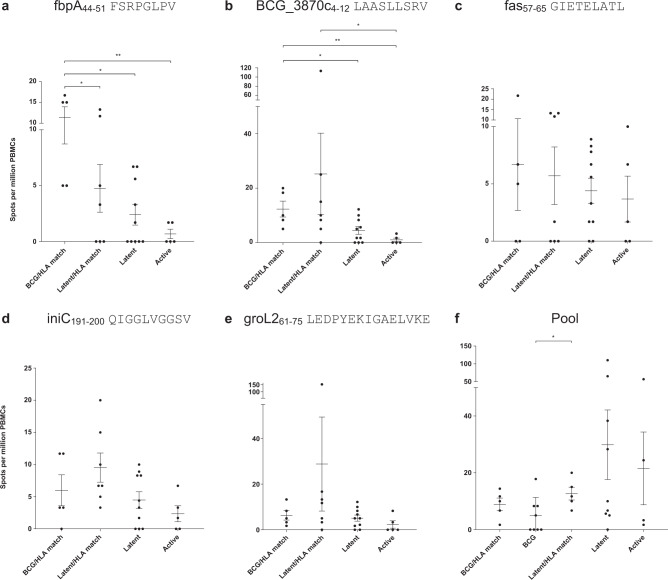
Peptide-specific responses of IFNγ secreting PBMCs from patient groups. ELISpots of PBMCs from 14 BCG-vaccinated individuals HLA-matched (*n* = 5), BCG-vaccinated individuals non-HLA matched (*n* = 8), latently infected individuals HLA-matched (*n* = 7), latently infected individuals non-HLA matched (*n* = 10 in (**a**, **b**, **c**, **d**, **e**); *n* = 9 in (**f**) and tuberculosis active patients (*n* = 5 in (**a**, **b**, **c**, **d**, **e**); *n* = 4 in (**f**) were used. Cells were stimulated with 2μg/ml of individual or pooled peptides or 20μg/ml PPD, or left unstimulated, with 3 × 10^5^ cells per well in duplicates. Cells were stimulated with (**a**) fbpA_44–51_ FSRPGLPV, (**b**) BCG_3870c_4–12_ LAASLLSRV, (**c**) fas_57–65_ GIETELATL, (**d**) iniC_191–200_ QIGGLVGGSV, (**e**) groL2_61–75_ LEDPYEKIGAELVKE and (**f**) a pool of peptides containing these five sequences and mmpL12_396–403_ AGCTLLIR and atpA_333–340_ KANDISAY (a peptide identified with a Peaks score of 14.54 hence excluded from the analysis). Each dot represents the results from each peptide. The error bars show the mean and standard errors of the mean. Statistical analysis performed with Two-tailed Mann–Whitney U test, and statistically significant differences between groups are represented as *p < 0.05, **p < 0.01.

The Ag85A peptide, fbpA_44–51_ FSRPGLPV, showed the highest response in the BCG-vaccinated HLA-matched group (mean = 11.34 spots per million PBMCs). There was a statistically significant difference between the responses in the BCG-vaccinated HLA-matched group, compared with the responses in the *M.tb* infected HLA-matched group (*p* = 0.048), the non-HLA-matched group (*p* = 0.0143) and the TB disease group (*p* = 0.0079) (Fig. 3a).

The glfT2 peptide, BCG_3870c_4–12_ LAASLLSRV, showed the highest response in the *M.tb* infected HLA-matched group (mean = 25.23 spots per million PBMCs). However, the responses in the BCG-vaccinated HLA-matched group were significantly different from the responses in the *M.tb* infected non-HLA matched (*p* = 0.041) and active TB groups (p = 0.0079). The responses in the *M.tb* infected HLA-matched group were different from those in the active TB group (*p* = 0.0189) (Fig. 3b). Responses to the fatty acid synthase peptide, fas_57–65_ GIETELATL, the isoniazid inducible gene protein iniC peptide, iniC_191–200_ QIGGLVGGSV, and the 60 kDa chaperonin 2 peptide, groL2_61–75_ LEDPYEKIGAELVKE were not different between groups (Fig. 3c–e, respectively). Responses to the pool of peptides composed of the five previous peptides, mmpL12_396–403_ AGCTLLIR and atpA_333–340_ KANDISAY, were significantly higher in the *M.tb* infected HLA-matched compared to the BCG-vaccinated non-HLA matched (Fig. 3f). Therefore, although the IFNγ-ELISpot responses were generally low for all groups, significant differences were observed for several individual peptides and the peptide pool in BCG-vaccinated individuals.

### Selected antigens conferred significant protection when delivered in combination

Peptides identified by mass spectrometry (Fig. 1), originating from mycobacterial antigens that are expressed in infected macrophages (Fig. 2), validated by spectral match validation (Supplementary Fig. 3) and ELISpot (Fig. 3) were selected to be evaluated as vaccine candidates. To determine whether these antigens could offer protective efficacy against TB disease, we produced viral vectors expressing each antigen (Supplementary Fig. 4). These antigens were evaluated as vaccine candidates alone or in combination in a murine aerosol *M.tb* challenge experiment.

**Fig. 4:**
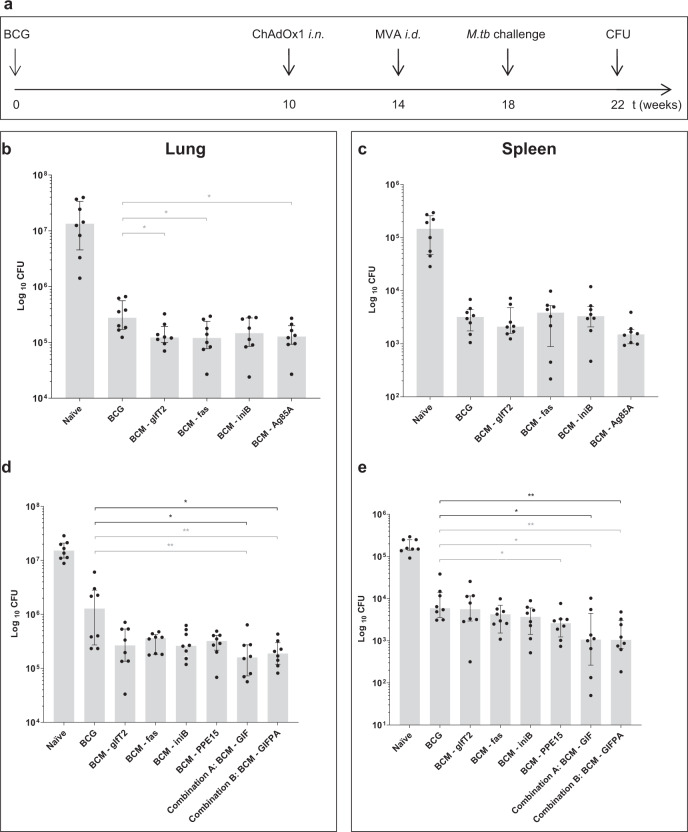
Protective efficacy of selected antigens alone or in combination, against a challenge with an aerosol of *M.tb* Erdman. **a** Experimental design. All groups of CB6F1 mice were vaccinated with BCG, intradermally. Ten weeks later, mice were boosted with intranasal ChAdOx1, followed by intradermal MVA, 4 weeks later, the BCM schedule. A group of mice was vaccinated with BCG only and another group left unvaccinated. Four weeks after BCM, all mice were challenged with an aerosol of *M.tb* Erdman (100 CFU per animal). Four weeks later, animals were culled, lung and spleen harvested and CFU counted. CFU from lung (**b**) and spleen (**c**), from the first experiment comprising of BCM expressing single antigens (glfT2, fas, iniB or Ag85A). CFU from lung (**d**) and spleen (**e**), from the second experiment consisting of BCM expressing single antigens (glfT2, fas, or iniB) or a combination of these three antigens (GIF) or a combination of GIF plus Ag85A and PPE15 (GIFPA). CFU, colony forming units, *n* = 8. Each symbol represents one animal, the error bars represent the median and the columns represent the interquartile range. Kruskal–Wallis followed by Dunn’s multiple comparison test (black bars) as well as Mann–Whitney U test (grey bars) were used to assess significance, *p < 0.05, **p < 0.01.

The BCM vaccination schedule consists on a BCG vaccination (B), followed by a boost of a replication-deficient chimpanzee adenovirus, ChAdOx1 (C) and a second boost of replication-deficient modified Vaccinia virus Ankara, MVA (M), expressing the antigen of interest (Fig. 4a).^24^ Two challenge experiments were performed. In the first experiment viral vectors expressing Ag85A were used as a positive control (Fig. 4b, c). Mice vaccinated with BCM regimens with individual antigens showed a significant lower median CFU compared to BCG vaccination alone in the lung, using Mann–Whitney U test (MW): BCG vs. BCM-glfT2 (*p* = 0.0207), BCG vs. BCM-fas (*p* = 0.0281) and BCG vs. BCM-Ag85A (*p* = 0.0207), although not significantly different for BCG vs. BCM-iniB or when using Kruskal–Wallis followed by Dunn’s multiple comparison tests (KW-DM), in lung and spleen (Fig. 4b, c).

In the second experiment, viral vectors expressing PPE15, recently described as a new protective antigen against TB,^25^ were used as a positive control. The BCM regimen with BCM-PPE15 showed a significant lower median CFU compared to BCG vaccination alone (MW *p* = 0.0148) in the spleen but not in the lung and not with KW-DM. The other individual antigens were not significantly different compared to BCG vaccination alone both with MW or KW-DM. However, when combining the three antigens glfT2, iniB and fas (GIF), even at 2.5 fold lower dose per antigen, the efficacy of this regimen was significantly higher in the lung, compared to BCG alone (MW *p* = 0.0095, KW-DM *p* = 0.0144) (Fig. 4d), and in the spleen (MW *p* = 0.0148, KW-DM *p* = 0.0141) (Fig. 4e). When combining GIF with PPE15 and Ag85A (GIFPA) at the same dose, the significance is maintained in the lung, compared to BCG vaccination alone, (MW *p* = 0.0042, KW-DM *p* = 0.0261) (Fig. 4d), and in the spleen (MW *p* = 0.0019, KW-DM *p* = 0.0081) (Fig. 4e). Therefore, new pooled antigens identified by immunopeptidomics protected mice from *M.tb* disease in a murine challenge model.

## Discussion

We have identified MHC-I and MHC-II bound peptides in macrophages infected with *M. bovis* BCG. We have successfully identified 94 mycobacterial peptides presented by MHC-II and 43 presented by MHC-I, from 76 and 41 antigens, respectively. Three new antigens were expressed alongside others in viral vectors, and evaluated as vaccine candidates alone or in combination in a murine aerosol *M.tb* challenge model. When delivered in combination, these antigens conferred significant protection in the lungs and spleen compared with BCG vaccination alone. This demonstrates proof-of-concept for an unbiased approach to discover new candidate antigens.

The new vaccine antigens are glfT2, fas and iniB. The galactofuranosyltransferase, glfT2, identified in this study as BCG_3870c, contains 637 amino acids and is 100% identical to the glfT2 of *M.tb* (Rv3808c). This is a bifunctional transferase required for the biosynthesis of *M.tb* arabinogalactan,^26^ part of the mycolyl–arabinogalactan–peptidoglycan (mAGP) complex of the cell wall which is essential for mycobacterial viability^27^ and its crystal structure has been resolved.^28^ Interestingly, this antigen was listed in the first report of peptide identification from *M.tb* using mass spectrometry.^13^

The fatty acid synthase, fas in *M. bovis* BCG (Rv2524c in *M.tb*), is a very large protein containing 3069 amino acids in both species, with 99.97% identity. Fas, also known as FAS-I, is a eukaryotic‐like enzyme that is involved in fatty acid biosynthesis in mycobacteria.^29–31^ FAS-I is an essential multifunctional enzymatic complex, that catalyses the de novo synthesis of fatty acids from acetyl‐CoA, contributing to the virulence of *M.tb* by elongating fatty acids.^30^ The crystal structure of this enzyme has been resolved.^30^ In fact, several enzymes of the polyketide synthase family were identified in this study (pks12 presented by MHC-I, pks2 and pks8 presented by MHC-II), thus suggesting that these may also be relevant targets for vaccine development. Moreover, the transfer of mycolic acids produced by the FAS-I and FAS-II complexes to arabinogalactan synthesised partially by glfT2 is catalysed by the Antigen 85 complex.^31,32^ In this study, the vaccine GIFPA containing a combination of antigens that included fas, glfT2, Ag85A and PPE15 and iniB, showed the highest efficacy.

The isoniazid inducible gene protein iniB is a 479 amino acid long protein identified for the first time by mass spectrometry in 2002.^13^ In the same paper the authors described the identification of peptides from hsp65 (groEL2) and glfT2 (Rv3808c), both also identified in our study. That study identified three nested sequences of the isoniazid inducible gene protein iniB from *M.tb* (Rv0341) through pan-HLA class-I immunoprecipitation (Rv0341_33–42_,GLIDIAPHQI; Rv0341_33–44_, GLIDIAPHQISS; Rv0341_33–45_, GLIDIAPHQISSV).^13^ The same nested sequence was identified 15 years later as an *M.tb* peptide associated with soluble HLA-E (Rv0341_33–47_, GLIDIAPHQISSVAA).^14^ We have identified Isoniazid inducible gene protein iniC peptide sequence iniC_191–200_ QIGGLVGGSV in two experiments (Supplementary Table 2). Moreover, the Rv0341 (iniB) protein shares 27.96% identity with iniC, and the sequence iniC_191–200_ has five amino acids in common with Rv0341. Although we have not identified peptides from iniB, we found peptides from iniC, suggesting that this family (encoded in an iniBAC operon) may be co-expressed. These genes were highly expressed by MTC bacteria in infected macrophages.^33^ Even though the antigen alone did not confer protection compared to BCG, significance was obtained when in combination with other antigens (Fig. 4).

Most antigens presented by MHC-I and MHC-II were associated with the biosynthesis and transport of lipids and were located at the cell membrane. These include but were not limited to the mycobacterial membrane protein Large (mmpL) family, which we have identified mmpL12, mmpL14 and mmpL3, presented by MHC-I molecules. The mmpLs are transporters involved in the export of lipids across the cell wall, and have been implicated in drug resistance and are drug targets.^34,35^ These proteins are conserved across mycobacteria and most of their functions are unknown.^34^ MmpL3, which is essential for viability,^36^ has received much interest as a drug target.^35^ Its crystal structure has been recently resolved,^35^ it is involved in the transport of trehalose monomycolate (TMM) across the cell wall, a precursor of trehalose dimycolate TDM, which is a major glycolipid involved in mycobacterial virulence^37^ by activating matrix metalloproteinases.^38^ TMM is exported from the plasma membrane to the periplasmic space by mmpL3 and sent to the Ag85 complex that metabolises TMM into TDM.^34^ MmpL3 has also been implicated in the import of heme.^34^

In this study we identified nested peptides presented by MHC-II, originating from Heparin-binding hemagglutinin, hbhA in *M. bovis* BCG, which is 199 amino acid long, and has 100% identity to Rv0475 of *M.tb* H37Rv and is an important virulence factor of *M.tb*.^39^ This antigen is a well-known immunogen and vaccine candidate^40,41^ that is able to induce multifunctional CD4^+^ T cells in HIV and non-HIV co-infected TB patients.^42^ It induces a subset of CD4^+^ T cells with cytolytic functions,^41^ and it is a known adhesin^43^ that is involved in bacterial agglutination and has been implicated in the dissemination of *M.tb*.^44^ It has been found in different intracellular locations, participates in the formation of lipid inclusions and can bind charged lipids specifically to 4,5-phosphatidylinositol diphosphate.^45^

We also have also identified a series of nested peptides presented by MHC-II originating from GroL2. The GroL2 of *M. bovis* BCG (also known as 60 kDa chaperonin 2, protein CPN60-2, 65 kDa antigen, heat shock protein 65, cell wall protein A and antigen A^46^) is a well-known human^47^ and veterinarian^48^ vaccine candidate against TB as well as against head and neck cancer,^49^ that has been widely studied as a DNA vaccine in mice.^50^ GroL2 has 100% identity to the well-known GroEL2 of *M.tb*, 94.83% identity with GroEL of *M.leprae*, 93.37% identity with GroEL of the saprophyte *M. smegmatis* and 47.16% identity with human heat shock proteins. This 540 amino acid long antigen, activates dendritic cells^51^ and macrophages through the TLR4/NF-κB signalling pathway^52^ and induces strong CD4^+^ T cell responses.^47^ Due to high conservation between human and mycobacterial heat shock proteins, peptides presented by MHC molecules might induce cross-reactivity of T cells that would break tolerance that could lead to autoimmunity.^53^ Therefore, GroL2 epitopes should be carefully considered when designing vaccines against TB, even though they are presented by MHC-II molecules in infected macrophages.

The PE/PPE family were identified for the first time in the *M.tb* genome sequence^29^ and have gathered much interest from the community as potential vaccine candidates.^25^ Here we used PPE15 (Rv1039c), as a control antigen, alone and in combination with other antigens. Even though PPE15 alone conferred protection compared to BCG in the spleen but not in the lung, it has been shown to be protective in previous studies^25^ and in our study was able to confer significant protection when used in combination with other antigens (Fig. 4). We observed significant protective efficacy when the new antigens were combined, suggesting immunological synergy between these antigens. The immunogenicity of these antigens alone or in combination, the mechanisms by which protective efficacy is improved, and immune correlates of protection which are still largely unknown, remains to be addressed in future studies.

In our study we identified PE/PPE family members, such as the PPE50 and PE-PGRS50. Interestingly, peptide pools from PPE50/PPE51 induced significant numbers of IFNγ secreting T cells by ELISpot in PBMCs from *M.tb* infected individuals compared to patients with TB disease and PE-PGRS50 induced significant numbers of IFNγ secreting T cells from patients with TB disease compared to *M.tb* latently infected individuals.^3^ The implication of the MHC presentation of peptides from PPE50 and PE-PGRS50 on the outcome of *M.tb* infection or TB disease are yet to be determined. Many other relevant genes were also identified in this study, which could also be of interest as new vaccine candidates, including ppsD, sseB, carB, groS and rplV, among others.

The pool of peptides used in IFNγ-ELISpots showed a difference between responses in BCG-vaccinated non-HLA-matched subjects and HLA-matched volunteers with latent *M.tb* infection. In fact, a higher response of the peptide pool in *M.tb* latently infected individuals may reflect the contribution of the BCG_3870c_4-12_ LAASLLSRV peptide, as it showed a significantly higher response in PBMC from HLA-matched latently infected individuals compared to patients with TB disease. Also, the groL2_61–75_ LEDPYEKIGAELVKE peptide showed a high response in PBMC from HLA-matched, latently infected individuals. These responses with the single peptides may result in a higher overall response in HLA-matched, latently infected individuals against a pool of peptides. The contribution of the groL2 and BCG_3870 peptides may have hindered the effect of the other individual peptides on the pool. Interestingly, the single peptides fbpA_44–51_ FSRPGLPV and BCG_3870c_4–12_ LAASLLSRV, showed a significantly higher response in PBMC from HLA-matched, BCG-vaccinated volunteers compared to non-HLA-matched, *M.tb* infected subjects and subjects with TB disease, suggesting potential recognition of these epitopes by BCG-vaccinated individuals.

We propose a new combination of techniques based in immunopeptidomics to discover, analyse and test new vaccine candidates against tuberculosis. We have identified multiple new MHC class-I and class-II antigens, tested three new antigens alone or in combination to show efficacy, driving forward the discovery of new vaccines that are desperately needed to control this global pandemic.

## Methods

### Cells and bacteria

Human primary monocytes were obtained from leukapheresis cones from the local blood transfusion service. Human monocyte derived macrophages were produced as described previously.^54^ PBMCs were obtained from healthy UK adults aged 18–55 who participated in vaccination studies approved by the Oxfordshire Research Ethics Committee.^55^ PBMC from BCG-vaccinated individuals were obtained from a previous clinical trial NCT00480714 (TB003),^55^ or ongoing clinical trials: ClinicalTrials.gov Identifier NCT02380508 (TB038) and NCT02709278 (TB041). Samples from latently infected individuals were obtained from a previous clinical trial NCT00456183 (TB007)^56^ and samples from TB disease patients were obtained from a previous study.^57^ Methods using samples collected from human volunteers were conducted in accordance with the ethical principles of the Declaration of Helsinki, Good Clinical Practice, and local regulatory requirements. All volunteers provided informed consent for study participation.

THP-1 monocytes (ATCC TIB-202) were induced into phagocytic macrophage-like cells by stimulation with 20 nM Phorbol 12-Myristate Acetate (PMA, Sigma-Aldrich, UK), overnight. Cytokine treatments included 50 ng/mL IFNγ, 50 ng/mL TNFα (both from Immuno Tools, Germany) and 1 μg/mL anti-IL10, (clone JES3-9D7, BD Biosciences, UK) post PMA activation. HLA genotype of the cells and detailed allelic information is shown in Supplementary Table 1.

HeLa cells (ATCC CCL-2.1) were used for ChAdOx1 and MVA infections to monitor the expression of each antigen, by western blot.

For in vitro infection, BCG Montreal (ATCC 35735),^58^ containing pEGFP cloned under the control of mycobacterial 19 kDa promoter^59^ was kindly provided by Dr Rajko Reljic from St George's University of London, whereinafter designated as BCG-GFP. BCG-GFP was grown in medium containing Middlebrook's 7H9 broth (BD Biosciences, UK), supplemented with 10% OADC enrichment (BD Biosciences, UK), 0.2% glycerol and 0.05% tyloxapol at 37 °C, in aerobic conditions, on a shaker at 200 rpm.^54^ BCG-GFP was used to determine the rate of infection of the THP-1 infection experiments, by flow cytometry.

For BCG mouse vaccinations, BCG Pasteur (ATCC 35734) was grown in 7H9 broth (BD Biosciences, UK) containing 0.05% Tween 80 (Sigma-Aldrich, UK) and 10% ADC (Sigma-Aldrich, UK).

For *M.tb* mouse challenges, *M.tb* Erdman K01 (TMC107) was obtained from BEI resources, Manassas, USA.

### Macrophage infection

Bacterial cultures in exponential growth phase were centrifuged at 2851*g* for 10 min, washed in phosphate-buffered saline (PBS), and resuspended in the desired culture medium without antibiotics. To dismantle bacterial clumps, the bacterial suspension was subjected to 15 min of ultrasonic bath. Residual clumps were removed by 1 min centrifugation at 931*g*. Single-cell suspension was verified by fluorescence microscopy. THP-1 cells were infected with BCG-GFP, alive or heat-killed, at a Multiplicity of Infection (MOI) of 10, with or without pre-treatment with IFNγ and anti-IL10 (Supplementary Fig. 1a and Table 1) for 4 h at 37 °C with 5% CO_2_. Following internalisation, cells were washed with PBS and resuspended in appropriate culture medium without antibiotics.^54^ One or 7 days post-infection, cells were harvested, lysed and total proteins collected (Supplementary Fig. 1a).

### Flow cytometry and western blot

Macrophages were harvested with a cell scraper and fixed with 4% paraformaldehyde for 20 min, followed by cell staining with antibodies specific for human HLA-ABC, dilution 1:100 (clone W6/32, BioLegend, UK, Cat. No. 311418) or HLA-DR dilution 1:25 (clone L243, BioLegend UK, Cat. No. 307606), for 30 min. Determination of the rate of infection was performed based on the percentage of GFP positive cells, on fixed cells 24 h post-infection, after washing several times with PBS to remove extracellular bacteria. Samples were run on an LSR II flow cytometer and the data analysed using FlowJo (TreeStar Inc, Ashland, USA).

HeLa cells infected with ChAdOx1 and MVA cloned with each selected antigen were used detect antigen expression by western blot, using the iBind Western System (Invitrogen, UK). Antigens were detected with a primary Histidine Tag antibody, diluted 1:1000 BioRad, Cat. No. MCA1396, clone AD1.1.10, followed by a secondary polyclonal antibody, Goat Anti-Mouse IgG Fc (Alkaline Phosphatase), dilution 1:1000 (Abcam, UK, Cat. No. ab98710).

### Isolation of HLA-bound peptides

An equivalent of 1 × 10^8^ cells were lysed in 1 ml lysis buffer (0.5% Igepal, 150 mM NaCl, 50 mM Tris, pH 8.0, supplemented with complete™ protease inhibitor cocktail (Roche)). HLA complexes were immunoprecipitated using 1 mg monoclonal antibody L243 against HLA-DR per 1 × 10^8^ cells followed by 1 mg monoclonal antibody W6/32 against pan-HLA class-I complexes (GE healthcare) cross-linked to Protein A Sepharose beads using dimethyl pimelimidate (DMP, Sigma) (Supplementary Fig. 1b). Lysates were incubated overnight with L243 beads followed by W6/32 beads. Beads were subsequently washed with 10 column volumes of 2 × 150 mM NaCl in 50 mM Tris, 1 × 450 mM NaCl in 50 mM Tris and 50 mM Tris buffer without salt. Peptides bound to the HLA groove were released with 5 mL 10% acetic acid. The HLA-bound peptides were further purified by HPLC (Ultimate 3000, Thermo Scientific) on a ProSwift RP-1S 4.6 × 50 mm column (Thermo Scientific) by applying a linear gradient of 2–35% (v/v) acetonitrile in 0.1% (v/v) formic acid in water over 10 min (Supplementary Fig. 1c). Alternating fractions that did not contain larger complex components were pooled into two final fractions, concentrated and kept at −80 °C prior to MS analysis.

### Mass spectrometry and data analysis

Peptides were suspended in 20 μl buffer A (1% acetonitrile, 0.1% TFA in water) and analysed by nUPLC-MS/MS using an Ultimate 3000 RSLCnano System coupled with an Orbitrap Fusion Lumos Tribrid mass spectrometer (Thermo Scientific) or a TripleTOF 5600 (Sciex) (first experiment only).^12^ Nine microliters of each sample were injected and trapped onto a 3 μm particle size 0.075 mm × 150 mm Acclaim PepMap RSLC column at 8 μl/min flowrate. Peptide separation was performed at 40 °C by applying a 1 or 2 h linear gradient of 3–25% (v/v) acetonitrile in 0.1% (v/v) formic acid, 5% DMSO in water at a flowrate of 250 μl/min on a 2 μm particle size, 75 μm × 50 cm Acclaim PepMap RSLC column. For HLA class II samples, a linear gradient of 5–30% (v/v) acetonitrile was applied. Peptides were introduced to a Fusion Lumos mass spectrometer (Thermo Scientific) via an Easy-Spray source at 2000 V. The ion transfer tube temperature was set to 305 °C. Measurement of precursor peptides was performed with a resolution of 120,000 for full MS (300–1500 m/z scan range) at an AGC target of 400,000. Precursor ion selection and fragmentation by high-energy collisional dissociation (HCD at 28% collision energy for charge state 2–4, 35% for charge state 1) was performed in TopSpeed mode at an isolation window of 1.2 Da for singly to quarterly charged ions at a resolution of 30,000 and an AGC target of 300,000 in the Orbitrap for a cycle duration of 2 s. Singly charged ions were acquired with lower priority.^8^

MS data were analysed with Peaks 8^60^ (Bioinformatics Solutions) for identification of peptide sequences (Supplementary Fig. 1d). Spectra were matched to all reviewed 20997 human SwissProt entries from 09/11/2017 combined with *Mycobacterium bovis* (strain BCG/Pasteur 1173P2) was produced based on UniProt proteomes.^61^ The results were filtered using a score cut-off of −lg10P = 15. The searches were performed with the following parameters: no enzyme specificity, no static and variable modifications, peptide tolerance: ±5ppm and fragment tolerance: ±0.03 Da.

Human sequences were disregarded from the analysis. The PSMs of mycobacterial origin obtained were analysed following a pipeline consisting of: (1) A Peaks score cut-off of 15 was applied to all samples (Fig. 1d and Supplementary Fig. 1e); (2) Identical BCG sequences identified in every sample or containing incorrect identification were considered false positives and were excluded (Fig. 1d); (3) A stringent Blast analysis was performed on every peptide sequence using the DeBosT script. Peptides with two or more amino acids different from human sequences were considered non-human and identified as BCG peptides (Supplementary Fig. 1f). Peptides with higher netMHC rank were prioritised (Supplementary Fig. 1g). All the combinations of I/L amino acids were also considered to exclude potential human sequences. MHC-I peptides were divided based on their presence in more than one sample (8) or not (35). When found in more than one sample, peptides observed in different experiments (5) were separated from peptides seen in the same experiment (3). The MHC-I peptides found in one sample (35) were divided into different peptide sequences found on the same antigen (6) or peptide sequences found in one antigen only (29). MHC-II peptides were divided following the same criteria; however, the peptides found in more than one sample included nested peptides (20) which is a common feature of HLA-bound peptides,^62^ or found in one sample (74). When found in more than one sample and nested, peptides observed in different experiments (17) were separated from peptides seen in the same experiment (3). The MHC-II peptides found in one sample were divided into different peptide sequences found on the same antigen (6) or peptide sequences found on one antigen only (68) (Fig. 1d). Selected peptides sequences were confirmed by spectral match validation against a synthetic peptide (Supplementary Fig. 1h and 3). Final lists of MHC class-I and MHC class-II peptides were generated (Supplementary Tables 2 and 3, respectively).

### DeBosT script

Blast searches (National Library of Medicine, https://blast.ncbi.nlm.nih.gov) of putative mycobacterial sequences were performed using a batch script, available upon request. Mycobacterial sequences with less than two amino acid differences compared to human sequences were excluded from downstream analysis.

### Synthetic peptides

Synthetic peptides were synthesised by Mimotopes, UK, GeneScript, USA and Peptide Protein Research, UK. Peptides were dissolved in DMSO to 5 mM for spectral match validation and biological validations (ELISpot).

### Viral vector generation

ChAdOx1 and MVA expressing each antigen were cloned using GeneArt Technology (ThermoFisher Scientific, UK). Four mammalian codon-optimised antigens (fasD1, fasD2, glfT2, iniB) and flanked by a Kozak consensus sequence, a tPA leader sequence, a GS linker at the 5′-end and a PK tag, Histidine tag and STOP codon at the 3′ end, were cloned into a GeneArt entry vector and then recombined into ChAdOx1 or MVA destination plasmids as previously described.^63,64^ ChAdOx1 and MVA expressing Ag85A and PPE15 were produced as previously described.^24,25^ Construct sequences are available upon request.

### Mice

Six to nine weeks old female CB6F1/Crl mice were purchased from Charles River, UK. All procedures were performed in accordance with the UK Animals (Scientific Procedures) Act 1986 under project license number P9804B4F1 granted by the UK Home Office and received ethical approval from the Local Ethical Review Committee at the University of Oxford. Hybrid CB6F1 mice were used to include a broader MHC presentation compared to inbred parental C57BL/6 and BALB/c strains.

### Immunisations

The vaccination regimen was composed of a BCG prime, followed by an adenovirus (ChAdOx1) initial boost and a MVA second boost, expressing each antigen to be evaluated as a new vaccine candidate. This regimen, designated as BCM, has been validated as the best method to induce protection in a mouse *M.tb* aerosol challenge model.^24,25^

Groups of eight mice were vaccinated with 50 μL 4 × 10^5^ CFU BCG Pasteur (ATCC 35734) intradermally (i.d.), followed by intranasal (i.n.) booster vaccination 10 weeks later, with 1 × 10^8^ infectious units (IU) of ChAdOx1.glfT2, ChAdOx1.fasD1 mixed with ChAdOx1.fasD2, ChAdOx1.iniB, ChAdOx1.PPE15, ChAdOx1.Ag85A alone or in combination in 50 μL, followed by intradermal vaccination 4 weeks later, with 5x10^6^ plaque forming units (pfu) of MVA containing the same antigens in 50 μL. For vaccines containing multiple antigens, 0.4 × 10^7^ IU of ChAdOx1 and 2 × 10^6^ pfu of MVA containing each antigen were administered.

### *M.tb* challenge experiments

Four weeks after the last vaccination, mice were challenged with an *M.tb* aerosol. Aerosol challenge was performed using a Biaera AeroMP-controlled nebuliser (Biera technologies; Hagerstown, USA) contained in a Biosafety level 3 TCOL isolator as previously described.^24,25^ Animals were loaded in nose-only restrainers and exposed to aerosolised *M.tb* Erdman K01 (TMC107) (BEI resources; Manassas USA), prepared at 1 × 10^6^ CFU/mL in the nebuliser. The challenge protocol consisted of a 10 min run, followed by a 5 min purge, at an airflow 12 L/min, and pressure 20 psig. Mice were infected with approximately 50–100 CFU, confirmed 24 h post- challenge in two mice per experiment.

Four weeks post-challenge, lungs and spleens of mice were harvested for mycobacterial quantification. Organs were placed in reinforced homogenisation tubes (Stretton-scientific, UK) containing 1 ml of PBS and homogenised using Precellys-24 (Stretton-scientific) at 5500 rpm for 20 s. Dilutions were prepared in PBS and plated in duplicate on modified 7H11 agar plates (APHA).^65^ Plates were incubated at 37 °C and counted 5 weeks later (Fig. 4 and Supplementary Fig. 1j).

### Transcriptional profiling intracellular *M. bovis* BCG

THP-1 cells were infected at an MOI of 10:1 for 4 h at 37 °C with 5% CO_2_. After 4 h, the cells were washed with PBS and then incubated in RPMI without antibiotics for a further 20 h. Mycobacterial RNA was harvested using the GTC/Trizol differential lysis method and purified using RNeasy columns (Qiagen) with DNase treatment.^33^ Mycobacterial RNA yield and quality was assessed using the NanoDrop One Spectrophotometer (Thermo Scientific) and Agilent 4200 TapeStation (Agilent Technologies). Libraries for RNA sequencing were prepared from three (2ug total RNA) biological replicates of intracellular *M. bovis* BCG after rRNA depletion (Ribo-Zero, Illumina) using the NEBNext Ultra II kit (New England Biolabs), and sequenced on an Illumina NextSeq500 sequencer. Paired-end reads were mapped to the *M. bovis* BCG Pasteur 1173P2 genome using STAR (v2.6.0c) and normalised using Relative Log Expression (RLE) in DESeq2 (v1.20.0).^66^ Transcript abundance was estimated by ranking the average expression ratio for each gene relative to all genes.

### Ex vivo Interferon-gamma ELISpot assay

PBMCs isolated from whole blood and ELISpots were performed as previously described.^55^ Responses to selected individual peptides or a pool of peptides, 2 µg/mL each peptide, identified in this study were assessed. Responses were corrected by subtracting the number of spots from the unstimulated cells. Results were reported as spot-forming cells (SFC) per million PBMCs (Fig. 3 and Supplementary Fig. 1i).

### Statistical analyses

Statistical analysis was performed on GraphPad Prism 8. Analysis of data sets was performed using two-tailed Mann–Whitney U test or Kruskal–Wallis followed by post-hoc tests, for multiple comparisons.

### Reporting summary

Further information on research design is available in the Nature Research Reporting Summary linked to this article

## Supplementary information

Supplementary Information

Reporting Summary

## Data Availability

Fully annotated RNA-seq data have been deposited in ArrayExpress; accession number E-MTAB-8057. The mass spectrometry data that supports the findings of this study are available at PRIDE, identifier PXD015646 (fourth experiment). Other experimental data is available from the corresponding author upon request.
