# Supplementary material for: Identification of antigens presented by MHC for vaccines against tuberculosis
Source: NPJ Vaccines. 2020 Jan 3;5:2. doi: 10.1038/s41541-019-0148-y (PMC6941960; doi:10.1038/s41541-019-0148-y)
Supplement: Supplementary file 1 — Supplementary Information [file 41541_2019_148_MOESM1_ESM.pdf]

Supplementary data for

# Identification of antigens presented by MHC for vaccines against tuberculosis

Paulo Bettencourt[1], Julius Müller[1*], Annalisa Nicastri[2*], Daire Cantillon[3*], Meera Madhavan[1], Philip D. Charles[2], Carine B. Fotso[1], Rachel Wittenberg[1], Naomi Bull[1], Nawamin Pinpathomrat[1], Simon J. Waddell[3], Elena Stylianou[1], Adrian V. S. Hill[1], Nicola Ternette[1,2#], Helen McShane[1#]

1. Jenner Institute, University of Oxford, Oxford OX3 7DQ, UK.
2. Target Discovery Institute, University of Oxford, Oxford OX3 7FZ, UK.
3. Department of Global Health and Infection, Brighton and Sussex Medical School, University of Sussex, Brighton, BN1 9PX, UK.

[*] - Equal contributions.

[#] - Equal contributions.

**Corresponding author:**
Dr Paulo Bettencourt
The Jenner Institute, University of Oxford
Old Road Campus Research Building
Roosevelt Drive, Oxford OX3 7DQ

E-mail: paulo.bettencourt@ndm.ox.ac.uk
Tel: +44 (0)1865 617100

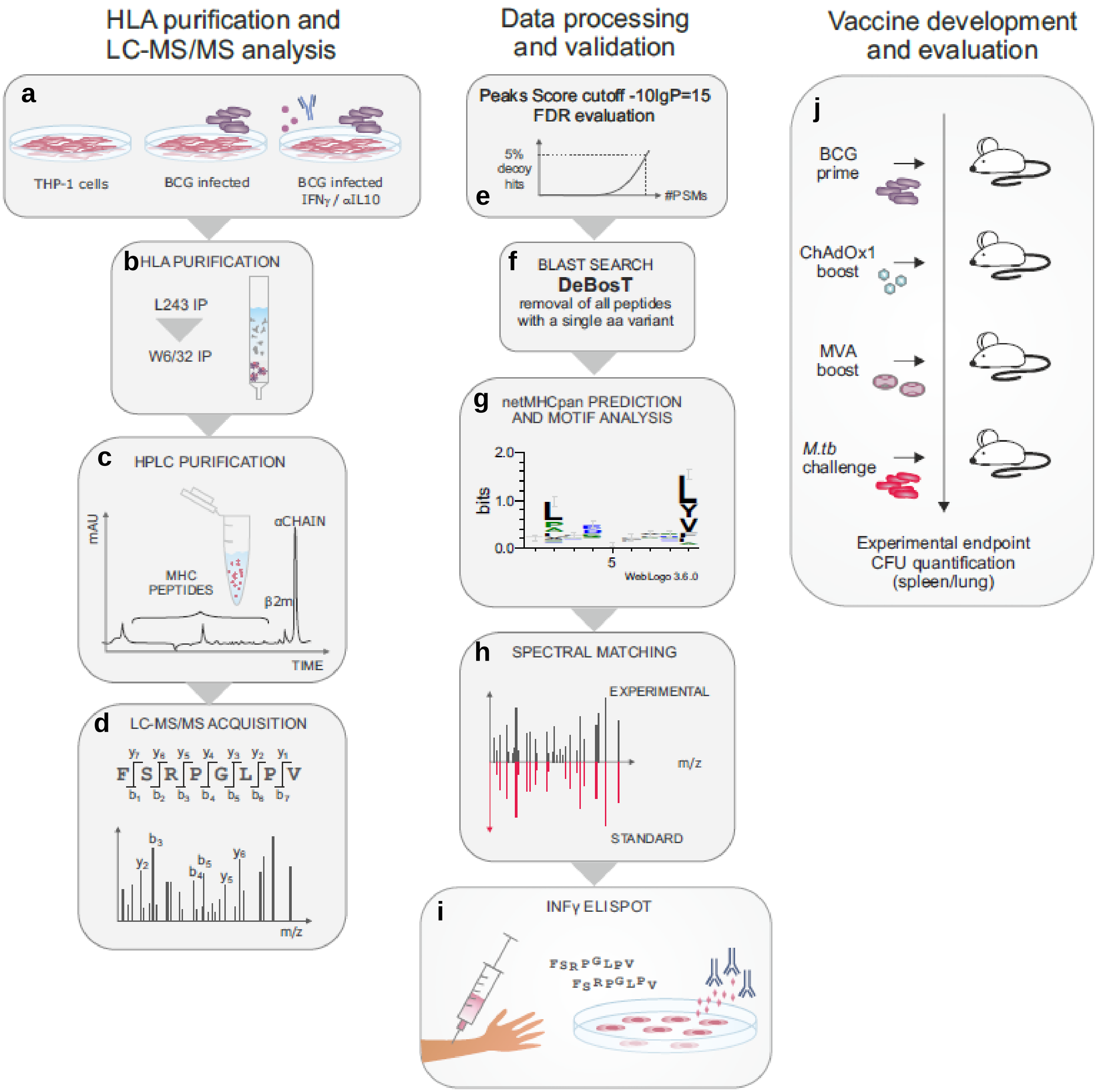


**Supplementary Figure 1** Immunopeptidomics pipeline for MHC class-I and class-II peptide identification by mass spectrometry and bioinformatics. **a** to **d** Sample preparation and mass spectrometry. **e** to **i** Data analysis and peptide identification. **a** THP-1 cells were differentiated with 20nM PMA to macrophage-like cells and infected with BCG-GFP, alive or heat-killed, at a MOI 10, with or without pre-treatment with IFNγ and anti-IL10. **b** THP-1 cells were harvested, lysed and total proteins collected. HLA molecules were purified by immunoprecipitation with monoclonal antibody against HLA-DR (clone L243), followed by monoclonal antibody against pan-HLA class-I (clone W6/32). **c** Peptides bound to the HLA groove were released from the HLA groove after 10% acetic acid denaturation of the α-chains and β-2-microglobulin, and separated by HPLC, using a C18 preparative column and a gradient of acetonitrile in formic acid 0.1% (v/v). **d** The HPLC fraction containing peptides were concentrated and injected in the LC-MS/MS for PSM identification. **e** PSMs with Peaks score higher than 15 were selected. **f** PSMs identified as *Mycobacterium bovis* BCG were blasted against the human proteome using the DeBost algorithm. Peptides with one amino acid different from human were excluded. Peptides with two or more amino acids different from human were selected. **g** Peptides with higher netMHC rank were prioritized. **h** Selected peptides sequences were confirmed by spectral match validation**. i** Immunogenicity evaluated by IFN-gamma ELISpot against PBMC from BCG-vaccinated volunteers, *M.tb* latently infected subjects or patients with tuberculosis disease. **j** Vaccine efficacy evaluated in murine aerosol *M.tb* challenge experiment.

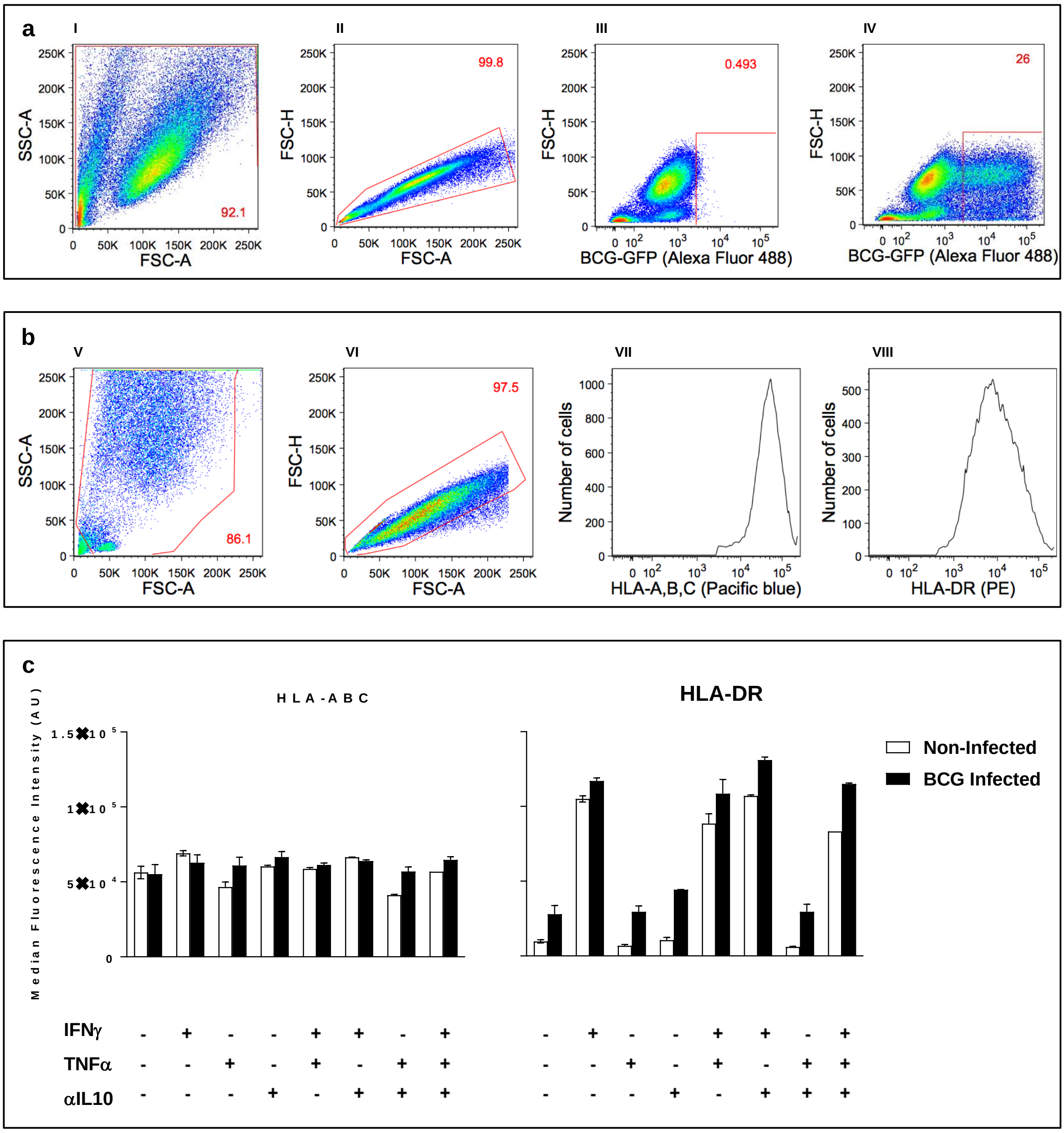


**Supplementary Figure 2** Infection and MHC activation of human macrophages. **a** Gating strategy to determine the rate of infection in THP-1 cells. Cells were acquired and plotted on side scatter-area (SSC-A) versus forward scatter-area (FSC-A) to define the population, 92.1% of events (I). Cells were subsequently gated for singlets using forward scatter-height (FSC-H) versus FSC-A, 99.8% of cells (II). Gated cells were plotted using FSC-H versus Alexa Fluor 488, to determine the percentage of GFP-negative events, 0.493%, representing uninfected cells (III) and compared to GFP-positive events, 26%, cells infected with a BCG expressing GFP (IV). In this representative example, non-infected sample contained 86,000 events and the infected sample contained 89,000 events. For each experiment, triplicates were used to calculate the rate of infection. **b** Gating strategy to quantify MHC activation in human monocyte derived macrophages. Single-stained and unstained mouse beads were used to calculate compensations. Samples containing 50,000 cells were acquired, plotted and gated for singlets as described in **a** (V and VI, respectively). Gated events were plotted in histograms, number of cells versus Pacific blue, to determine the distribution of the fluorescence intensity of HLA-A,B,C (VII). Gated events were also plotted in histograms, number of cells versus PE, to determine the distribution of the fluorescence intensity of HLA-DR (VIII). The fluorescence intensity of the histograms represent the surface marker expression. **c** Expression of HLA-A,B,C and HLA-DR in human monocyte derived macrophages stimulated with IFNγ, TNFα, anti-IL10, alone or in combination and infected with BCG. The columns represent the average and the error bars represent the standard deviation. Each condition was tested in duplicate or triplicate except the triple positive stimulated non-infected cells, with one replicate. No statistical test was performed due to low number of replicates.

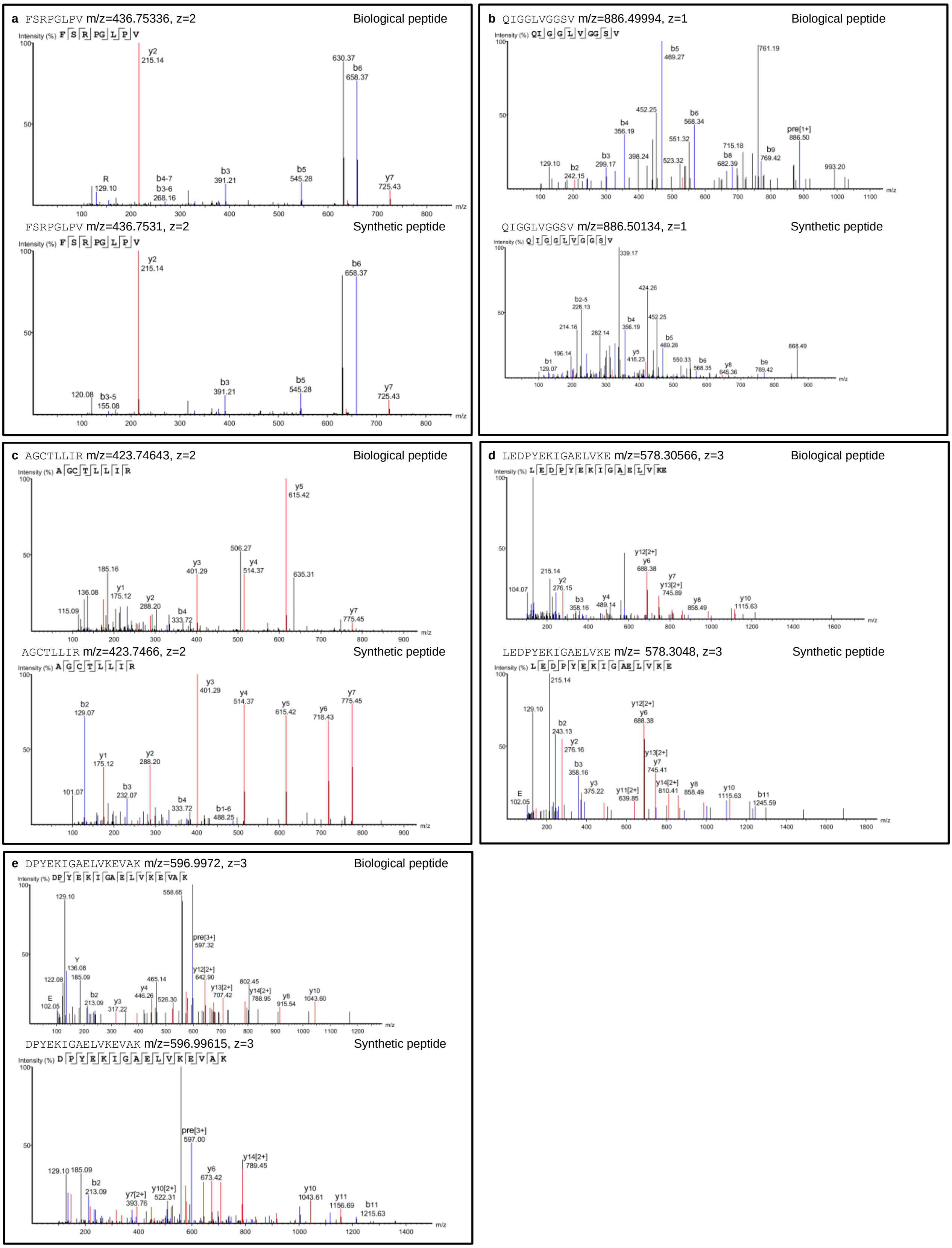

a FSRPGLPV m/z=436.75336, z=2
Biological peptide
FSRPGLPV m/z=436.7531, z=2
Synthetic peptide
b QIGGLVGGSV m/z=886.49994, z=1
Biological peptide
QIGGLVGGSV m/z=886.50134, z=1
Synthetic peptide
c AGCTLLIR m/z=423.74643, z=2
Biological peptide
AGCTLLIR m/z=423.7466, z=2
Synthetic peptide
d LEDPYEKIGAELVKE m/z=578.30566, z=3
Biological peptide
LEDPYEKIGAELVKE m/z= 578.3048, z=3
Synthetic peptide
e DPYEKIGAELVKEVAK m/z=596.9972, z=3
Biological peptide
DPYEKIGAELVKEVAK m/z=596.99615, z=3
Synthetic peptide
Intensity (%)
m/z

**Supplementary Figure 3** Spectral Match Validation. HLA-associated peptide sequences were synthesized and measured by LC-MS/MS under identical conditions as the experimentally identified peptide sequences. Both the biological and the synthetic peptides were plotted relative to each other to illustrate the spectral match. Fragment ions are labelled in the spectra and the respective molecular fragment is indicated in the peptide sequence above each spectrum. Ions are labelled as follows: b: N-terminal fragment ion; y: C-terminal fragment ion; pre: precursor peptide ion. Immonium ions are indicated using the regarding single amino acid letter code (i.e. Y, E, R). Charge state is shown for each signal in brackets, and ions are singly charged if no charge state shown. The mass to charge ratio [m/z] of the precursor peptide ion and the charge state is shown for each spectrum. Fragment mass spectra for five peptides validated. Examples of MHC-I validated peptides: **a** fbpA$_{44\text{-}51}$ FSRPGLPV, **b** iniC$_{191\text{-}200}$ QIGGLVGGSV, **c** mmpL12$_{396\text{-}403}$ AGCTLLIR, and MHC-II validated nested peptides: **d** groL2$_{61\text{-}75}$ LEDPYEKIGAELVKE. **e** groL2$_{63\text{-}78}$ DPYEKIGAELVKEVAK.

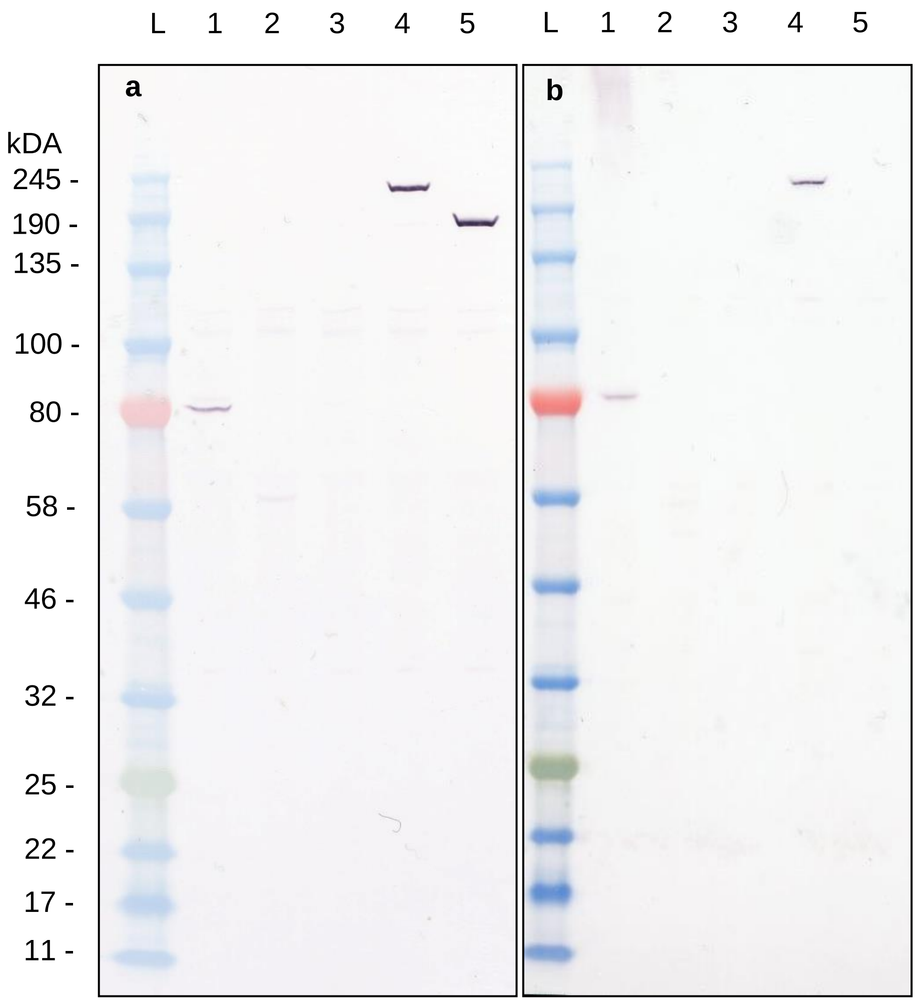


**Supplementary Figure 4** Expression of antigens by viral vectors. Expression of selected antigens in HeLa cells infected ChAdOx1 (**a**) and MVA (**b**) expressing each antigen. L – Ladder Broad Range (11-245 kDa) NEB #P7712. Lane 1 - glfT2; 2 - Rv0341; 3 - Infected with irrelevant ChAdOx1; 4 - fas D1; 5 - fas D2.

**Supplementary Table 1** HLA Genotyping of THP-1 cells

| Locus | Allele | Allelle | Interpreted | | |
|---|---|---|---|---|---|
| HLA-A | *02:01-604 | - | A | 2 | |
| HLA-B | *15:11/15/209N/305/367 | - | B | 75 | |
| HLA-C | *03:03/13/18/20N-316N | - | Cw | 9 | |
| HLA-DRB1 | *01:01/05/07-14/16-73 | *15:01/12/13/16/20/22-128 | DR | 1 | 15 |
| HLA-DRB3 | | | | | |
| HLA-DRB4 | | | | | |
| HLA-DRB5 | *01:01-05/08N/10N/14-18 | | DR | 51 | |
| HLA-DQB1 | *06:02/14/47/68/70/72-147 | *05:01/07/11/12/18/20-63 | DQ | 6 | 5 |
| HLA-DPB1 | *04:02 | *02:01 | DP | | |

The Histocompatibility and Immnunogenetics Report was produced by the Transplant Immmunology & Immunogenetics, Oxford Transplant Centre, The Churchill Hospital, Oxford, UK.

**Supplementary Table 2** MHC-I peptide identification

| Peptide identification | | Antigen identification | | | HLA-peptide binding | | Experimental details | | |
|---|---|---|---|---|---|---|---|---|---|
| **Sequence** | **n** | **UniProt** | **Name** | **Symbol** | **netMHC best hit** | **netMHC best score** | **Experiment number** | **Sample** | **Peaks score (-10*log10(Pvalue))** |
| *Peptides identified in different experiments* | | | | | | | | | |
| FSRPGLPV | 8 | A1KQD8/ | Diacylglycerol acyltransferase/mycolyltransferase Ag85A/ | fbpA/ | HLA-C*03:13 | 2.3986 | 4 | CYT HKBCG B | 24.81 |
| | | A1KJU9/ | Diacylglycerol acyltransferase/mycolyltransferase Ag85B/ | fbpB/ | | | 4 | CYT HKBCG A | 22.84 |
| | | A0A0H3M0R3 | Secreted antigen 85-c fbpC (85C) | fbpC | | | 3 | CYT HKBCG | 22.77 |
| QIGGLVGGSV | 10 | A0A0H3M1A8 | Isoniazid inductible gene protein iniC | iniC | HLA-A*02:01 | 14.7788 | 4 | CYT HKBCG B | 23.29 |
| | | | | | | | 2 | DAY7 A | 17.89 |
| | | | | | | | 4 | CYT LIVEBCG A | 16.49 |
| IGAGAIAVAA | 10 | A0A0H3MFF6 | Probable conserved integral membrane protein [second part] | BCG_2410 | HLA-C*03:03 | 12.5346 | 4 | CYT LIVEBCG A | 18.47 |
| | | | | | | | 2 | DAY7 A | 15.91 |
| LDSRQFERY | 9 | A0A0H3M738 | Polyketide synthetase mbtD | mbtD | HLA-B*15:15 | 5.9146 | 2 | DAY1 A | 18.45 |
| | | | | | | | 1 | DAY7 B | 15.37 |
| GIGAGAAVL | 9 | A0A0H3MCA7 | Probable conserved transmembrane protein | BCG_3967 | HLA-C*03:13 | 2.2331 | 4 | CYT HKBCG B | 18.11 |
| | | | | | | | 2 | DAY7 A | 15.62 |
| *Peptides identified in more than 1 sample* | | | | | | | | | |
| LAASLLSRV | 9 | A0A0H3MAQ2 | Galactofuranosyl transferase | BCG_3870c | HLA-C*03:03 | 1.0334 | 1 | DAY1 B | 28.16 |
| | | | | | | | 1 | DAY1 A | 21.06 |
| AGCTLLIR | 8 | A0A0H3M689 | Probable conserved transmembrane transport protein mmpL12 | mmpL12 | HLA-B*15:15 | 89 | 3 | NOCYT HKBCG | 24.98 |
| | | | | | | | 3 | CYT LIVEBCG | 22.93 |
| ASSVFAPAL | 9 | A0A0H3M6D2 | Putative conserved integral membrane transport protein | BCG_2476c | HLA-C*03:13 | 0.3975 | 4 | CYT LIVEBCG B | 21.49 |
| | | | | | | | 4 | NOCYT HKBCG B | 18.66 |
| | | | | | | | 4 | NOCYT LIVEBCG B | 17.25 |
| *Peptides identified in the same antigen* | | | | | | | | | |
| PALMVLLGR | 9 | A0A0H3M5L6 | Probable conserved transmembrane transport protein mmpL14 | mmpL14 | HLA-A*02:01 | 55 | 1 | DAY1 A | 19.01 |
| PAIAGATF | 8 | A0A0H3M5L6 | | | HLA-B*15:11 | 3.1917 | 2 | DAY1 A | 16.62 |
| GIETELATL | 9 | A0A0H3MFR6 | Probable fatty acid synthase fas | fas | HLA-A*02:01 | 2.6087 | 3 | NOCYT LIVEBCG | 18.48 |
| ADLVVIVGGA | 10 | A0A0H3MFR6 | | | HLA-A*02:01 | 10.5071 | 2 | DAY1 A | 15.68 |
| VTVNATPD | 8 | A0A0H3M7L7 | Probable polyketide synthase pks12 | pks12 | HLA-B*15:11 | 65 | 4 | NOCYT LIVEBCG A | 16.06 |
| VNGPSAVVV | 9 | A0A0H3M7L7 | | | HLA-C*03:13 | 6.804 | 4 | NOCYT LIVEBCG B | 15.77 |
| *Peptides identified once* | | | | | | | | | |
| LHVEQLQTI | 9 | A0A0H3M9J8 | Probable conserved transmembrane protein | BCG_3501c | HLA-C*03:13 | 4.0392 | 2 | DAY1 A | 23.68 |
| LFAFLDDHS | 9 | A0A0H3M7I5 | Putative transposase | BCG_2830 | HLA-A*02:01 | 38 | 2 | DAY7 A | 23.28 |
| ALAAAAAGVT | 10 | A0A0H3M9Q7 | Probable Sn-glycerol-3-phosphate-binding lipoprotein ugpB [first part] | ugpBa | HLA-A*02:01 | 3.9356 | 4 | CYT HKBCG A | 21.36 |
| LAADALVLKV | 10 | A0A0H3MCT0 | Transposase for insertion sequence element IS1081 | BCG_2532c | HLA-C*03:03 | 3.611 | 2 | DAY7 B | 19.45 |
| LAPPPADPA | 9 | A0A0H3M8Z9 | Possible resuscitation-promoting factor rpfA | rpfA | HLA-C*03:03 | 2.641 | 2 | DAY7 A | 19.45 |
| LAEIYQGEV | 9 | A0A0H3M6G7 | Methionine synthase | metH | HLA-C*03:03 | 4.9093 | 2 | DAY1 A | 19.15 |
| AADAAHRAGV | 10 | A0A0H3MCA7 | Probable conserved transmembrane protein | BCG_3967 | HLA-C*03:03 | 5.5597 | 2 | DAY1 A | 19.08 |
| TLHEVPVL | 8 | A0A0H3MAQ0 | Uncharacterized protein | BCG_3864c | HLA-A*02:01 | 2.5974 | 4 | CYT LIVEBCG A | 18.5 |
| TMIEYYLHE | 9 | A0A0H3MFH1 | Uncharacterized protein | BCG_2427c | HLA-A*02:01 | 6.55 | 2 | DAY7 A | 18.25 |
| EDCHFCSQSG | 10 | A1KJ05 | Biotin synthase | bioB | HLA-B*15:15 | 95 | 2 | DAY7 A | 17.68 |
| EHADWIVDIGP | 11 | A0A0H3M4F7 | UvrABC system protein A | uvrA | HLA-A*02:01 | 80 | 2 | DAY7 B | 17.58 |
| LSIPSVAIP | 9 | A0A0H3M5L1 | PPE family protein | PPE24 | HLA-C*03:03 | 12.3553 | 2 | DAY7 A | 17.47 |
| APEAVDLLVQLA | 12 | A0A0H3MFU7 | Conserved hypothetical alanine leucine valine rich protein | BCG_2582c | HLA-A*02:01 | 32.4138 | 2 | DAY1 A | 17.31 |
| TIGGVADHLAT | 11 | A1KM20 | 1-deoxy-D-xylulose-5-phosphate synthase | dxs | HLA-A*02:01 | 42.75 | 2 | DAY1 B | 17.1 |
| ADGTDGGKGGNGG | 13 | A0A0H3M617 | PE-PGRS family protein | PE_PGRS27 | HLA-B*15:15 | 97.5 | 4 | NOCYT LIVEBCG A | 17.07 |
| PSGSGTPLVSY | 11 | A0A0H3M520 | Probable fatty-acid-CoA ligase fadD24 | fadD24 | HLA-B*15:15 | 19.9571 | 2 | DAY1 B | 16.89 |
| AQVRASVTY | 9 | A1KF53 | R2-like ligand binding oxidase | BCG_0270 | HLA-B*15:15 | 0.0538 | 2 | DAY1 B | 16.76 |
| WDCAAVNV | 8 | A0A0H3MC94 | Uncharacterized protein | BCG_1242c | HLA-C*03:03 | 50.3846 | 3 | CYT HKBCG | 16.59 |
| GPAFAALS | 8 | A0A0H3MGQ7 | Phenolpthiocerol synthesis type-I polyketide synthase ppsD | ppsD | HLA-B*15:11 | 19.3043 | 4 | CYT LIVEBCG B | 16.51 |
| ITNVSIPAI | 9 | A0A0H3M207 | PPE family protein | PPE8 | HLA-C*03:03 | 0.5682 | 2 | DAY7 A | 16.41 |
| PATASFAYA | 9 | A0A0H3MA86 | PPE family protein | PPE4 | HLA-C*03:03 | 23.942 | 4 | CYT LIVEBCG A | 16.18 |
| GGGLSSPVI | 9 | A0A0H3M376 | PE-PGRS family protein | PE_PGRS14 | HLA-C*03:13 | 9.0088 | 2 | DAY7 A | 15.86 |
| PSSGRITVAGV | 11 | A0A0H3MDE4 | Probable 'component linked with the assembly of cytochrome' transport transmembrane ATP-binding protein ABC transporter cydD | cydD | HLA-A*02:01<br>HLA-B*15:15 | 70 | 3 | CYT HKBCG | 15.86 |
| AAIEERLSR | 9 | A0A0H3M1D1 | Uncharacterized protein | BCG_0405c | HLA-B*15:11 | 7.0108 | 4 | NOCYT HKBCG A | 15.7 |
| DPVSILDVRW | 10 | A0A0H3MF71 | Sulfurtransferase | sseB | HLA-C*03:13 | 6.8816 | 2 | DAY7_B | 15.57 |
| RTLFRVPF | 8 | A0A0H3M2S6 | Conserved transmembrane transport protein mmpL3 | mmpL3 | HLA-C*03:13 | 7.4497 | 3 | CYT HKBCG | 15.38 |
| DYSDAGLYL | 9 | A0A0H3M5F4 | Aminotransferase | BCG_1241 | HLA-B*15:11 | 2.9928 | 2 | DAY1 A | 15.34 |
| PATLAAALP | 9 | A0A0H3M9A8 | Probable membrane protein | BCG_1061c | HLA-C*03:03 | 58.4091 | 2 | DAY7 A | 15.24 |
| HPVLVDRFLED | 11 | A0A0H3MCS3 | Carbamoyl-phosphate synthase large chain | carB | HLA-C*03:13 | 68.75 | 4 | CYT HKBCG A | 15.01 |

**Supplementary Table 3** MHC-II peptide identification

| Peptide indentification | | Antigen identification | | | HLA-peptide binding | | Experimental details | | |
|---|---|---|---|---|---|---|---|---|---|
| Sequence | n | Accession | Name | Symbol | netMHCpan best hit | netMHCpan best score | Experiment number | Sample ID | Peaks score (-10*log10(Pvalue)) |
| *Nested peptides identified in different experiments* | | | | | | | | | |
| **K**APAKKAAAK | 10 | A1KFU9 | Heparin-binding hemagglutinin | hbhA | HLA-DRB5*01:01 | 44 | 3 | CYT HKBCG | 21.26 |
| **K**APAKKAAAKKVTQK | 15 | | | | HLA-DRB5*01:01 | 21 | 4 | NOCYT HKBCG A | 24.92 |
| AAAK**K**APAKKAAAKK | 15 | | | | HLA-DRB5*01:01 | 17 | 3 | CYT LIVEBCG | 17.24 |
| KKAAAK**K**APAKKAAAKK | 17 | | | | HLA-DRB5*01:01 | 12 | 4 | NOCYT LIVEBCG A | 28.56 |
| KKAAAK**K**APAKKAAAKK | 17 | | | | | | 4 | NOCYT LIVEBCG B | 17.03 |
| AKKAAPAKKAAPAKKAAAK**K** | 20 | | | | HLA-DRB5*01:01 | 12 | 4 | NOCYT LIVEBCG A | 17.73 |
| *Nested peptides* | | | | | | | | | |
| **DPYEKIGAELVK** | 12 | A1KFR2 | 60 kDa chaperonin 2 | groL2 | HLA-DRB1*01:01 | 12 | 4 | CYT HKBCG A | 68.08 |
| **DPYEKIGAELVK**EVA | 15 | | | | HLA-DRB1*01:01 | 13 | 4 | CYT HKBCG A | 46.93 |
| **DPYEKIGAELVK**EVAK | 16 | | | | HLA-DRB1*01:01 | 17 | 4 | CYT HKBCG A | 49.14 |
| E**DPYEKIGAELVK**E | 14 | | | | HLA-DRB1*01:01 | 16 | 4 | CYT HKBCG A | 45.4 |
| LE**DPYEKIGAELVK**E | 15 | | | | HLA-DRB1*01:01 | 16 | 4 | CYT HKBCG A | 30.75 |
| NGEE**YLILSARDVLA** | 15 | A1KPA9 | 10 kDa chaperonin | groS | HLA-DRB1*01:01 | 1.9 | 4 | CYT LIVEBCG A | 68.22 |
| **YLILSARDVLA** | 11 | | | | HLA-DRB1*01:01 | 0.9 | 4 | CYT LIVEBCG A | 41.23 |
| QGG**AGGMGGSGADNA** | 15 | A0A0H3M9Q3 | PE-PGRS family protein | PE_PGRS54 | HLA-DRB1*01:01 | 95 | 4 | NOCYT LIVEBCG A | 24.29 |
| GG**AGGMGGSGADNA** | 14 | | | | HLA-DRB1*01:01 | 95 | 4 | NOCYT HKBCG B | 15.83 |
| **AGGMGGSGADNA** | 12 | | | | HLA-DRB1*01:01 | 95 | 4 | NOCYT LIVEBCG A | 18.32 |
| **GAGGDGGDGANFASG** | 15 | A0A0H3M3G6 | PE-PGRS family protein | PE_PGRS23 | HLA-DRB1*01:01 | 95 | 4 | NOCYT LIVEBCG B | 16.03 |
| **GAGGDGGDGANFASG**GAGG | 19 | | | | HLA-DRB1*01:01 | 95 | 4 | NOCYT LIVEBCG A | 21.42 |
| *Peptides identified in more than 1 sample* | | | | | | | | | |
| KAK(+43.99)**GHLDAGAK** | 11 | A0A0H3MAB5 | Glyceraldehyde-3-phosphate dehydrogenase | gap | HLA-DRB5*01:01 | 75 | 4 | CYT LIVEBCG A | 31.54 |
| K(+43.99)AK**GHLDAGAK** | | | | | | | 4 | CYT LIVEBCG A | 27.16 |
| DEETLQGYITR | 11 | A0A0H3MCS3 | Carbamoyl-phosphate synthase large chain | carB | HLA-DRB1*01:01 | 65 | 2 | DAY1 | 21.07 |
| | | | | | | | 2 | DAY7 | 20.96 |
| *Peptides identified in the same antigen* | | | | | | | | | |
| AGANGVAVGAP | 11 | A0A0H3M617 | PE-PGRS family protein | PE_PGRS27 | HLA-DRB1*01:01 | 95 | 2 | DAY1 | 23.01 |
| AGGDGGAPGDGAN | 13 | A0A0H3M617 | PE-PGRS family protein | PE_PGRS27 | HLA-DRB1*01:01 | 95 | 4 | NOCYT LIVEBCG B | 17.49 |
| LQDM(+15.99)AILTGGQVIS | 14 | A1KFR2 | 60 kDa chaperonin 2 | groL2 | HLA-DRB1*01:01 | 8 | 4 | CYT HKBCG A | 35.36 |
| AQTGVYEDLLAAGVADPVK | 19 | A1KFR2 | 60 kDa chaperonin 2 | groL2 | HLA-DRB1*01:01 | 35 | 4 | CYT LIVEBCG A | 35.11 |
| KAPAKKAPAKASETSAAKGGS | 21 | A1KGI7 | 50S ribosomal protein L22 | rplV | HLA-DRB5*01:01 | 85 | 4 | NOCYT LIVEBCG B | 21.12 |
| PAKKAAAKAPA | 11 | A1KGI7 | 50S ribosomal protein L22 | rplV | HLA-DRB5*01:01 | 38 | 4 | CYT LIVEBCG A | 16.31 |
| *Peptides identified once* | | | | | | | | | |
| S(+14.02)GQIKTGAPARSE | 13 | A1KHG1 | Enolase | eno | HLA-DRB5*01:01 | 30 | 4 | CYT HKBCG A | 42.78 |
| ANRAELKALIASNLLGQNT | 19 | A0A0H3M8U4 | PPE family protein | PPE50 | HLA-DRB1*01:01 | 5 | 4 | CYT LIVEBCG A | 39.94 |
| DTNYHYLVPEIGPS | 14 | A1KHS4 | 5-methyltetrahydropteroyltriglutamate--homocysteine methyltransferase | metE | HLA-DRB1*01:01 | 6 | 4 | CYT HKBCG A | 31.61 |
| GAGGKAGLLFGSGGA | 15 | A0A0H3MB82 | PE-PGRS family protein | PE_PGRS13 | HLA-DRB1*01:01 | 85 | 4 | NOCYT LIVEBCG A | 28.99 |
| PGGGGGAGGDGGAANTDS | 18 | A0A0H3M903 | PE-PGRS family protein | PE_PGRS15 | HLA-DRB1*01:01 | 95 | 4 | NOCYT HKBCG B | 27.9 |
| AKVINISVT | 9 | A0A0H3MC87 | Possible secreted alanine and proline rich protease | BCG_3941c | HLA-DRB1*01:01 | 23 | 4 | NOCYT HKBCG A | 25.85 |
| APMRPPRS | 8 | A0A0H3MDJ5 | Possible integral membrane efflux protein efpA | efpA | | | 3 | CYT LIVEBCG | 25.82 |
| KLVANSLIK | 9 | A0A0H3M5J1 | Probable serine protease htrA | htrA | HLA-DRB5*01:01 | 1.5 | 4 | CYT HKBCG A | 21.53 |
| KAPAKKTPAKGAKSAPPKPA | 20 | A0A0H3MC68 | Possible histone-like protein hns | hns | HLA-DRB5*01:01 | 80 | 4 | NOCYT LIVEBCG B | 21.46 |
| AVDPYAIGVP | 10 | A0A0H3M602 | Probable dehydrogenase | BCG_1493 | HLA-DRB1*15:01 | 80 | 2 | DAY7 | 21.32 |
| GANGAPGTTSTSGGN | 15 | A0A0H3M1W6 | PE-PGRS family protein | PE_PGRS7 | HLA-DRB1*01:01 | 95 | 2 | DAY7 | 20.82 |
| RVGIVSNDP | 9 | A0A0H3M2X4 | Probable molybdopterin biosynthesis protein moeA1 | moeA1 | HLA-DRB1*01:01 | 75 | 4 | CYT HKBCG A | 20.64 |
| ALAILDTVGV | 10 | A0A0H3M5J7 | UDP-N-acetylmuramoyl-L-alanyl-D-glutamate--2,6-diaminopimelate ligase | murE | HLA-DRB1*01:01 | 4.5 | 4 | NOCYT HKBCG A | 20.61 |
| RVGIDAEAGPT | 11 | A0A0H3M564 | Histidinol dehydrogenase | hisD | HLA-DRB1*01:01 | 95 | 4 | CYT LIVEBCG A | 19.9 |
| GTGGNASATGT | 11 | A0A0H3MIA6 | PE-PGRS family protein | PE_PGRS53 | HLA-DRB1*01:01 | 95 | 4 | CYT HKBCG A | 19.84 |
| GGWLLGDGGAGGSG | 14 | A0A0H3M8Q1 | PE-PGRS family protein | PE_PGRS9 | HLA-DRB1*01:01 | 55 | 2 | DAY1 | 19.62 |
| TAELLGAMSEA | 11 | A1KM16 | Uroporphyrinogen decarboxylase | hemE | HLA-DRB1*01:01 | 36 | 4 | NOCYT LIVEBCG B | 19.55 |

*Continued in next page*

**Supplementary Table 3** MHC-II peptide identification - continued

| Sequence | n | Accession | Name | Symbol | netMHCpan best hit | netMHCpan best score | Experiment number | Sample ID | Peaks score (-10*log10(Pvalue)) |
|---|---|---|---|---|---|---|---|---|---|
| *Peptides identified once* | | | | | | | | | |
| AAIAAAGTTIVL | 12 | A0A0H3MAU4 | Probable conserved transmembrane protein | BCG_3906c | HLA-DRB1*01:01 | 11 | 2 | DAY7 | 19.15 |
| RLGVDLAEV | 9 | A0A0H3MIH3 | Probable acyl-CoA dehydrogenase fadE34 | fadE34 | HLA-DRB1*01:01 | 55 | 3 | CYT LIVEBCG | 19.13 |
| LGRGLLGLI | 9 | A0A0H3MDN2 | Signal recognition particle receptor FtsY | ftsY | HLA-DRB1*01:01 | 20 | 2 | DAY7 | 18.42 |
| TELDEVTNGLHP | 12 | A0A0H3M178 | Replicative DNA helicase | dnaB | HLA-DRB1*01:01 | 90 | 4 | CYT LIVEBCG A | 18.41 |
| EINSARLYV | 9 | A0A0H3M207 | PPE family protein | PPE8 | HLA-DRB1*15:01 | 28 | 3 | CYT HKBCG | 18.25 |
| KMAAAGCGRIVLNSRTQPTQKMRET | 25 | A1KQG0 | Phthioceranic/hydroxyphthioceranic acid synthase | pks2 | HLA-DRB5*01:01 | 43 | 4 | NOCYT HKBCG A | 18.11 |
| KPLEVVVFHHRPERYQFS | 18 | A0A0H3M332 | Probable transcriptional regulatory protein (Probably gntR-family) | BCG_0845c | HLA-DRB5*01:01 | 31 | 4 | NOCYT HKBCG B | 17.98 |
| DIPNISDEVLADL | 13 | A1KGE7 | DNA-directed RNA polymerase subunit beta | rpoB | HLA-DRB1*01:01 | 90 | 4 | CYT HKBCG A | 17.97 |
| GDYEGLWGL | 9 | A0A0H3M8U0 | PPE Family protein [first part] | PPE55a | HLA-DRB1*01:01 | 80 | 3 | NOCYT LIVEBCG | 17.95 |
| PGAPERMVQNT | 11 | A0A0H3M155 | Possible conserved membrane protein | BCG_0323 | HLA-DRB1*01:01 | 95 | 4 | NOCYT HKBCG B | 17.9 |
| PPVIAAVFRK | 10 | A0A0H3MJ54 | Putative lipase lipE | lipE | HLA-DRB5*01:01 | 2.5 | 4 | NOCYT LIVEBCG B | 17.81 |
| AAELSRDLDFRAR | 13 | A0A0H3M6E7 | Probable transmembrane serine/threonine-protein kinase j pknJ | pknJ | HLA-DRB1*15:01 | 65 | 4 | CYT HKBCG A | 17.7 |
| TPTPRNAAGA | 10 | A0A0H3MAK2 | Possible transcriptional regulatory protein | BCG_0526 | HLA-DRB1*01:01 | 95 | 4 | CYT HKBCG A | 17.52 |
| VTLASILPVLA | 11 | A0A0H3M5J6 | Possible nitrate/nitrite transporter narK2 | narK2 | HLA-DRB1*01:01 | 2.5 | 4 | NOCYT HKBCG A | 17.49 |
| PVKAKLAPVP | 10 | A0A0H3M812 | Possible transmembrane protein | BCG_0465c | HLA-DRB1*01:01 | 36 | 2 | DAY7 | 17.37 |
| HGGSGGNGGSGGSGGS | 16 | A0A0H3MHS0 | PE_PGRS50 protein | PE_PGRS50 | HLA-DRB1*01:01 | 95 | 4 | NOCYT HKBCG B | 17.25 |
| KADLAAEH | 8 | A0A0H3MJE7 | Putative ferredoxin-dependent glutamate synthase [NADPH] (Large subunit) gltB | gltB | | | 4 | NOCYT LIVEBCG B | 17.21 |
| HESALLVSLGGA | 12 | A0A0H3M4I3 | Possible long-chain acyl-CoA synthase | BCG_1721 | HLA-DRB1*01:01 | 28 | 4 | NOCYT LIVEBCG B | 17.11 |
| IARQRRRPR | 9 | A1KPR5 | Uncharacterized tRNA/rRNA methyltransferase BCG_3644c | BCG_3644c | HLA-DRB5*01:01 | 3 | 4 | NOCYT LIVEBCG A | 17.06 |
| VVFLAYSGQARWRDS | 15 | A0A0H3M9W9 | Conserved hypothetical alanine and arginine rich protein | BCG_2938 | HLA-DRB5*01:01 | 3 | 4 | NOCYT LIVEBCG B | 17.05 |
| IVVAVPVGPA | 10 | A0A0H3M5I7 | Uncharacterized protein | BCG_2160 | HLA-DRB1*01:01 | 11 | 4 | CYT HKBCG A | 16.85 |
| LPQDAIRWAALVALVAIGA | 19 | A0A0H3M9U0 | Possible integral membrane C-type cytochrome biogenesis protein dipZ | dipZ | HLA-DRB1*01:01 | 22 | 4 | CYT LIVEBCG A | 16.77 |
| GLVVWQIYRPGR | 12 | A0A0H3M0K6 | Probable conserved transmembrane protein | BCG_0082 | HLA-DRB1*15:01 | 4 | 4 | CYT HKBCG A | 16.44 |
| GGAGGIGGSAVLFGAGG | 17 | A0A0H3M4W3 | PE-PGRS family protein | PE_PGRS29 | HLA-DRB1*01:01 | 90 | 4 | NOCYT LIVEBCG A | 16.32 |
| KPNPIGVGLME | 11 | A0A0H3MBW3 | Probable conserved two-domain membrane protein | BCG_3788 | HLA-DRB1*01:01 | 80 | 4 | NOCYT HKBCG B | 16.32 |
| KGGTGKSTIAAALALTLAAGGRK | 23 | A0A0H3MIR0 | Probable anion transporter atpase | BCG_3738 | HLA-DRB5*01:01 | 4.5 | 4 | NOCYT HKBCG A | 16.29 |
| PTYAFQRQ | 8 | A0A0H3M4G9 | Probable polyketide synthase pks8 | pks8 | | | 3 | CYT LIVEBCG | 16.26 |
| APLLAELIRGGAALSRVRHPGD | 22 | A0A0H3MAM8 | Uncharacterized protein | BCG_3838 | HLA-DRB1*01:01 | 3 | 4 | CYT HKBCG A | 16.23 |
| DGALTGGMC | 9 | A1KM20 | 1-deoxy-D-xylulose-5-phosphate synthase | dxs | HLA-DRB1*01:01 | 75 | 4 | NOCYT LIVEBCG B | 16.22 |
| DITLPMPPRWTQV | 13 | A0A0H3M0J7 | Secreted proline rich protein MTC28 (PROLINE RICH 28 kDa ANTIGEN) | mtc28 | HLA-DRB1*01:01 | 45 | 2 | DAY7 | 16.1 |
| GTQAKLAVIRVP | 12 | A0A0H3M833 | Probable exported protease | BCG_2240c | HLA-DRB5*01:01 | 27 | 2 | DAY7 | 16.04 |
| TALMVVRIE | 9 | A0A0H3M2H3 | Possible two component sensor kinase | BCG_0897 | HLA-DRB5*01:01 | 20 | 4 | CYT HKBCG A | 15.84 |
| VTVAPAMT | 8 | A0A0H3MCS3 | Carbamoyl-phosphate synthase large chain | carB | | | 4 | NOCYT HKBCG A | 15.8 |
| PGLAGLQALQR | 11 | A0A0H3M9W7 | Putative cation transporter P-type atpase ctpJ | ctpJ | HLA-DRB1*01:01 | 6.5 | 4 | CYT LIVEBCG A | 15.77 |
| RLLVIALKHNVI | 12 | A0A0H3M6I2 | Dihydrolipoamide acetyltransferase component of pyruvate dehydrogenase complex | pdhC | HLA-DRB1*15:01 | 1 | 4 | CYT HKBCG A | 15.76 |
| GKPALLRPV | 9 | A0A0G2Q9D4 | Beta sliding clamp | dnaN_1 | HLA-DRB1*01:01 | 38 | 2 | DAY1 | 15.75 |
| VTSGVGVAGVGVGLLG | 16 | A0A0H3M793 | Probable oxidoreductase | BCG_0116 | HLA-DRB1*01:01 | 80 | 4 | NOCYT LIVEBCG B | 15.74 |
| AAHAEGAAGLA | 11 | A0A0H3M5P8 | PPE family protein | PPE28 | HLA-DRB1*01:01 | 75 | 2 | DAY7 | 15.66 |
| RAAARRIGTAASVSVFE | 17 | A0A0H3MA06 | Possible oxidoreductase | BCG_0234 | HLA-DRB1*01:01 | 24 | 3 | CYT LIVEBCG | 15.66 |
| PAPVVVEVDLLD | 12 | A0A0H3M8A6 | Fatty-acid-CoA ligase fadD26 | fadD26 | HLA-DRB1*15:01 | 80 | 2 | DAY7 | 15.61 |
| EGLLSYLPPQGQ | 12 | A1KP90 | Putative S-adenosyl-L-methionine-dependent methyltransferase BCG_3469 | BCG_3469 | HLA-DRB1*15:01 | 7 | 4 | CYT LIVEBCG A | 15.54 |
| YVRVGKNGPY | 10 | A0A0H3MA36 | DNA topoisomerase 1 | topA | HLA-DRB5*01:01 | 19 | 3 | CYT HKBCG | 15.41 |
| DGPLGGISPVGFKPEGV | 17 | A0A0H3MFP8 | Integral membrane indolylacetylinositol arabinosyltransferase embA | embA | HLA-DRB1*01:01 | 90 | 4 | NOCYT HKBCG A | 15.41 |
| HGLDEVVIGMPHRGRLNVLAN | 21 | A1KI36 | Multifunctional 2-oxoglutarate metabolism enzyme | kgd | HLA-DRB5*01:01 | 23 | 4 | NOCYT HKBCG B | 15.39 |
| PAKKAAAKRPATKAPAKKATARR | 23 | A0A0H3M7X4 | Probable DNA-binding protein HU homolog hupB | hupB | HLA-DRB5*01:01 | 29 | 4 | CYT HKBCG A | 15.36 |
| TPEQLSRFE | 9 | A0A0H3MFR6 | Probable fatty acid synthase fas | fas | HLA-DRB1*01:01 | 95 | 3 | NOCYT LIVEBCG | 15.35 |
| SGLLLGLDGFNA | 12 | A0A0H3M7N8 | PE-PGRS family protein | PE_PGRS3 | HLA-DRB1*01:01 | 5 | 4 | NOCYT HKBCG B | 15.25 |
| LASEMPECQAGIGVAAGQ | 18 | A0A0H3MA33 | Possible adenylate cyclase | BCG_1381c | HLA-DRB1*01:01 | 85 | 4 | NOCYT LIVEBCG B | 15.23 |
| LSLTPEDWIA | 10 | A0A0H3MC15 | Integral membrane indolylacetylinositol arabinosyltransferase embB | embB | HLA-DRB1*01:01 | 65 | 4 | CYT HKBCGA | 15.16 |
| GLGDHTTAVLSLTRAI | 16 | A0A0H3M3X1 | Probable conserved transmembrane protein | BCG_1520c | HLA-DRB5*01:01 | 39 | 4 | CYT HKBCG A | 15.08 |
| VHRTGPGPM | 9 | A0A0H3M485 | Probable enoyl-CoA hydratase echA5 | echA5 | HLA-DRB5*01:01 | 28 | 4 | NOCYT HKBCG A | 15.07 |

The common amino acids of nested peptides are presented in bold.
N/D – Not determined.

**Supplementary Table 4** Description of the HLA types of human samples used in this study

| Volunteer | HLA match | Time point |
|---|---|---|
| | *BCG Vaccinated* | |
| 39 | A2/Cw9/DR15/DR51 | D14 |
| 10 | A2 | D14 |
| 12 | DR15/DR51 | D14 |
| 38 | A2/DR15/DR51 | D14 |
| 41 | DR1 | D14 |
| 117 | A2 | D14 |
| | *Latently Infected Volunteers* | |
| 9 | DR15/DR51 | W52 |
| 11 | B75 | Screening |
| 14 | A2 | W52 |
| 16 | DR15/DR51 | W52 |
| 17 | A2/DR15/DR51 | W52 |
| 25 | DR51 | Screening |
| 28 | DR15/DR51 | Screening |

D14 - Day 14. W52 - Week 52.
